# Spatio-Temporal Variation in the Phyllospheric Microbial Biodiversity of *Alternaria Alternata*-Infected Tobacco Foliage

**DOI:** 10.3389/fmicb.2022.920109

**Published:** 2022-07-28

**Authors:** Yuan-feng Dai, Xiao-mao Wu, Han-cheng Wang, Wen-hong Li, Liu-ti Cai, Ji-xin Li, Feng Wang, Shafaque Sehar, Imran Haider Shamsi

**Affiliations:** ^1^Department of Plant Protection, Institute of Crop Protection, College of Agriculture, Guizhou University, Guiyang, China; ^2^Guizhou Provincial Academician Workstation of Microbiology and Health, Guizhou Academy of Tobacco Science, Guiyang, China; ^3^Bijie Tobacco Company, Bijie, China; ^4^Guizhou Institute of Plant Protection, Guizhou Academy of Agricultural Sciences, Guiyang, China; ^5^Guizhou Tobacco Company of CNTC, China National Tobacco Corporation, Guiyang, China; ^6^Zhejiang Key Laboratory of Crop Germplasm Resource, Department of Agronomy, College of Agriculture and Biotechnology, Zhejiang University, Hangzhou, China

**Keywords:** microbial community, *Alternaria alternata*, tobacco brown spot, high-throughput sequencing, microbial functional diversity, biolog-eco

## Abstract

Phyllospheric microbial composition of tobacco (*Nicotiana tabacum* L.) is contingent upon certain factors, such as the growth stage of the plant, leaf position, and cultivar and its geographical location, which influence, either directly or indirectly, the growth, overall health, and production of the tobacco plant. To better understand the spatiotemporal variation of the community and the divergence of phyllospheric microflora, procured from healthy and diseased tobacco leaves infected by *Alternaria alternata*, the current study employed microbe culturing, high-throughput technique, and BIOLOG ECO. Microbe culturing resulted in the isolation of 153 culturable fungal isolates belonging to 33 genera and 99 bacterial isolates belonging to 15 genera. High-throughput sequencing revealed that the phyllosphere of tobacco was dominantly colonized by Ascomycota and Proteobacteria, whereas, the most abundant fungal and bacterial genera were *Alternaria* and *Pseudomonas*. The relative abundance of *Alternaria* increased in the upper and middle healthy groups from the first collection time to the third, whereas, the relative abundance of *Pseudomonas, Sphingomonas*, and *Methylobacterium* from the same positions increased during gradual leaf aging. Non-metric multi-dimensional scaling (NMDs) showed clustering of fungal communities in healthy samples, while bacterial communities of all diseased and healthy groups were found scattered. FUNGuild analysis, from the first collection stage to the third one in both groups, indicated an increase in the relative abundance of Pathotroph-Saprotroph, Pathotroph-Saprotroph-Symbiotroph, and Pathotroph-Symbiotroph. Inclusive of all samples, as per the PICRUSt analysis, the predominant pathway was metabolism function accounting for 50.03%. The average values of omnilog units (OUs) showed relatively higher utilization rates of carbon sources by the microbial flora of healthy leaves. According to the analysis of genus abundances, leaf growth and leaf position were the important drivers of change in structuring the microbial communities. The current findings revealed the complex ecological dynamics that occur in the phyllospheric microbial communities over the course of a spatiotemporal varying environment with the development of tobacco brown spots, highlighting the importance of community succession.

## Introduction

Tobacco is an important economic crop cultivated extensively for its leaves, which are dried and processed primarily for smoking in pipes, cigarettes, and cigars (Wang et al., 2013). According to FAOSTAT (FAO, 2019), about 100 countries produce tobacco, but China undoubtedly remains the major producer accounting for 39.06% (2,392,090 metric tons) of the global tobacco production (Wang et al., 2014; Xu et al., 2014). Many fungal and bacterial diseases occur on tobacco leaves during the course of tobacco production (Chen et al., 2020), even during the curing process (Rotem, 1994). One of the most important foliar fungal diseases is tobacco brown spot, which is caused by *Alternaria alternata* that is prone to infect senescent leaves (Ishida and Kumashiro, 1988). Initially, tobacco brown spots appear at the leaf maturity stage and are mostly observed in the lower leaves during June in China (Xu et al., 1993; He et al., 1997), then spread from the lower leaves to the top (LaMondia, 2001; Hou et al., 2016). Under high humidity conditions and appropriate conditions for infection, the spores germinate on the leaf surface in 24 h, then germ tubes were produced and penetrate the leaf cells directly through walls or stomata (Slavov et al., 2004). At last, germinating spores release toxins which could cause brown spot disease of tobacco and some small necrotic spots could be noticed 48 h after inoculation (Kodama et al., 1990). The saprophytic nature of *A. alternata* allows it to remain in the soil on a very large amount of lignified plant debris, over winter as mycelium and survive for several months by invading dead plant tissues. Due to the short incubation period (5–8 days) under the right conditions, *A. alternata* spreads swiftly and infects the stems, transmitting the disease through seeds by colonizing pedicels and capsules in extreme cases, when long periods of rain occur during harvesting (Shew and Lucas, 1991).

A number of microbial communities are established in the phyllosphere including fungi, bacteria, viruses, protists, and archaea (Berlec, 2012; Vorholt, 2012), the structure of which is strongly shaped by a myriad of abiotic and biotic factors, including space (Rastogi et al., 2012; Rumakanta et al., 2017), growth season (Copeland et al., 2015; Ding and Melchner, 2016), geographical location (Whipps et al., 2008; Chen et al., 2020), plant species (Whipps et al., 2008; Knief et al., 2010; Wellner et al., 2011), disease severities (Luo et al., 2017, 2019), pesticide treatments (Qin et al., 2019; Chen X. et al., 2021), and agricultural management (Karlsson et al., 2017). Similarly, phyllospheric microbial communities of tobacco were strongly correlated with the plant's spatiotemporally varying agro-ecosystem. Alterations in the microbial community are always accompanied by changes in the metagenomic environment of the host, exhibiting the methods through which phytopathogens invade and redirect the host's resources and functions and the strategies adopted by hosts to respond against pathogen invasion (Whipps et al., 2008). Therefore, the comprehension of these complex interactions among microbial species at multifarious levels is required to predict how variations in microbial diversity will impact the functioning of an ecosystem (Langenheder et al., 2010). Fungal communities have been reported to differ with phases of curing, i.e., the yellowing phase, the color-fixing phase, and the stem-drying phase, and with their respective collection height/position on the plant, even exhibiting some differences in tobacco petioles and lamina during the tobacco curing process (Rotem, 1994). Previous studies show contradictory results of either increment or reduction in the abundance of fungal community and diversity of tobacco leaves infected by *Golovinomyces cichoracearum, Didymella segeticola*, and *A. alternata* (Guo et al., 2020; Huang et al., 2020; Xiang et al., 2020). Moreover, pesticide treatments have been documented to influence bacterial communities colonizing the tobacco phyllosphere (Chen X. et al., 2021; Liu et al., 2021).

To date, many methods have been described to analyze the phyllospheric microbial structure and biodiversity. Normally, culturing methods are economic but also time-consuming and can only detect <1% of microorganisms (Chen et al., 2018). Previously, pathogen identification could only be performed individually or in small groups. However, with the development of molecular sequencing, the next-generation sequencing of high-throughput technique has become the main research approach to microbial classification, proving to be ultrasensitive and precise for the indepth identification of culturable and non-culturable microbes and the simultaneous quantification of microbial community structure (Jayawardena et al., 2018; Mboowa et al., 2018). This technological advancement not only puts emphasis on the micro-ecology of distinct microbial taxa but also on the collective genetic composition of microbial communities belonging to different niches or “microbiomes” (Henry et al., 2021). Several host-associated microbiomics studies affirm that microbial bio-divergence is a feature that constitutes a portion of the host organism's extended phenology (Bruijning et al., 2020) with immense implications on the health, fitness, and evolution of the host. In view of its significance on the host's function and fitness, crop scientists aspire to model and navigate host–microbiome interactions (Bruijning et al., 2020; Henry et al., 2021). Reportedly, the community-level physiological profile technique (BIOLOG) has effectively been utilized to ascertain the bacterial community's functional diversity (Zhang et al., 2010). In earlier investigations, the profiling of the tobacco phyllosphere microbiome concentrated mainly on the biodiversity analysis of field-grown tobacco leaves sampled at a single stage, which was infected by *G. cichoracearum, D. segeticola*, and *A. alternata* (Guo et al., 2020; Huang et al., 2020). Although there is a huge void in our current knowledge regarding the epidemiology of brown leaf spot disease, whether it is antagonized or synergized by other phyllospheric microbial communities during different growth stages of tobacco as the dynamics of microbial diversity is known to partake significantly in the robustness and probability of getting disease among crops, either *via* competitive response toward pathogens or by instigating the innate immunity of plants against them (Douglas et al., 2020).

Consequently, in the present study, our goal was to distinguish the tobacco microbial communities and the dynamic variation in microbial communities and their biodiversity in three leaf growth stages and three stalk positions. Moreover, we were interested in the microbial functional diversity during tobacco growth phases and the development of tobacco brown spots, particularly in the symbiotic relationship between microbial community and environmental factors. For this, culture-independent isolation and high-throughput techniques were employed. Furthermore, the substrate utilization profiling (BIOLOG) technique was used to determine the utilization patterns of carbon sources. This study gives a new insight into phyllospheric microbial alteration in the spatiotemporally varying ecosystems during different growing stages and provides a theoretical basis for the precise prediction of diseases and precise pesticide application.

## Materials and Methods

### Meteorological Observations

To record information on the relative humidity, air temperature, wind speed, and rainfall, an automatic weather station (Beijing Xinhong Tec. IT, Co., Ltd., Beijing, China) was constructed consisting of a hygroscope (MC-KWS), a thermometer (MC-KWS), an anemoscope (MC-FX), a rain gauge (MC-YL), etc. The meteorological data storage system (MC-YL) was employed to acquire and process all measurements. The following are the collection dates: the first stage (August 8, 2020), the second stage (August 30, 2020), and the third stage (September 19, 2020).

### Site Description and Sample Planting

An experimental field site was selected in Bijie city (27°22'5”N,105°19'57”E), located in the northwest of Guizhou Province of China. The fields were planted with tobacco cv. Yunyan87 for almost 2 years. Seedlings were planted during April and the growth management was kept the same as other tobacco plants but devoid of pesticide treatments. The chosen experimental area measured 60 m^2^, which was divided into three plots (20 m^2^ each), and then subdivided into three rows (1.1 m space between the rows). Ten Yunyan87 seedlings were planted with a 0.55 m gap between two plants on the same row.

### Sampling and Processing of *N. tabacum* L. Leaves

Leaf positions were determined as upper (1~6th top leaf), middle (7~12th top leaf), and lower (13~18th top leaf), measuring from the top of the flue-cured tobacco plant (Supplementary Figure 1). When tobacco brown spot was first observed (about 100 days after planting), healthy and diseased leaves infected by *A. alternata* from the above-mentioned three leaf positions were collected randomly from three plots at the time of 100, 122, and 142 days after planting, respectively. Two kinds of leaves were clipped carefully from the plants using sterile hand shears and 20 g of the leaves were put into 50 ml plastic centrifuge tubes, separately. The scissors were sterilized with 70% ethanol for 30 s after cutting each piece of leaf. Upon arrival in the laboratory, the sample containing tubes were placed straightaway at 4°C prior to culturing and BIOLOG, or in the case of high-throughput sequencing at −80°C.

For designating codes to samples, the letter BJY87 was used for healthy leaves and BJB87 for diseased tobacco leaves, followed by the number 1 for the first time to collect leaves, 2 for the second time, 3 for the third time, and then followed by 1 for the first replicate, 2 for the second replicate, 3 for the third replicate, and finally followed by “U” for the upper, “M” for the middle, and “D” for the lower positions, respectively. For instance, BJY8711U were healthy leaves for the first replicate collected the first time from the upper position in the field, and BJB8722D were the diseased leaves for the second replicate collected the second time from the lower position (Supplementary Table 1).

### Isolation and Molecular Identification of Phyllospheric-Culturable Microorganisms

#### Isolation and Identification of Bacteria

Culturable bacteria were isolated by using plate coating. Bacteria were isolated on Luria-Bertani (LB) media or nutrient agar (NA). Afterward, 10 g of leaves were placed in conical flasks with 300 ml of sterilized water and shaken for 1 h at 200 rpm, then 30, 40, and 50 ml supernatants were spread on both LB and NA plates. Once the plates underwent dark conditions for 2–5 days at 28°C, colonies with the same morphology, color, and growth rates were isolated. Axenic cultures were stored in 40% glycerin with LB bacterial solution at −20°C (LB: 40% glycerin = 1:1). The bacterial DNA was extracted using TIANamp Bacteria DNA Kit (Tiangen Biotech Co. Ltd., Beijing, China) according to the operating steps. The amplification of the 16S rRNA gene of isolates was carried out based on the procedures described by Li et al. (2021). Resultant PCR products were electrophoresed on a 1% agarose gel and the execution of targeted fragment size (16S 1600 bp) purification was done using Gene JET (Thermo Fisher Scientific, Waltham, MA, United States), then sequenced and used as a query in a BLASTN search of the NCBI nr database1. Moreover, for the identification of the isolates, matches with identity values >98% were used.

#### Isolation and Identification of Fungi

The tissue separation method was adopted for culturable fungal isolation (Attitalla et al., 2010). Isolation was done either on potato dextrose agar (PDA) or alkyl ester agar (AEA) according to the method of Chen X. et al. (2021). Diseased and healthy tissues (5 × 5 mm pieces) were directly inoculated on both PDA and AEA plates. The remaining tissues were placed in the dark at 28°C for 5 days, and the hyphae were then ready to be transferred to a fresh plate. Pigment, growth rate, and morphological traits were the distinct criteria for fungal isolation. Purified cultures were stored at 4°C on PDA slants. Molecular identification was conducted according to the method of Huang et al. (2021). The PCR products were electrophoresed on a 1% agarose gel and the purification of the ITS 500 bp fragment was exercised using Gene JET (Thermo Fisher Scientific, Waltham, MA, United States). Subsequently, the fragments were then sequenced and used as a query in a BLASTN search of the NCBI nr database1. For the purpose of identification of each isolate, matches with identity values >98% were used.

### Extraction of DNA, Amplification, and Sequencing Process

DNA was extracted from the tobacco phyllosphere using an EasyPure Plant Genomic DNA Kit (TransGen Biotech, China) with the CTAB method. Qualitative detection of the DNA solution was carried out by 2% agarose gel electrophoresis, while the quantitative detection was determined through NanoDrop ND-2000 (Thermo Fisher Scientific, Waltham, MA, United States). Amplification of the hypervariable V3–V4 region of bacterial 16S rRNA genes was performed with the primers, 341F (5'-CCTACGGGRBGCASCAG-3') and 806R (5'-GGACTACNNGGGTATCTAAT-3') (Jeanette et al., 2012). The fungal ITS1-5F region was amplified using ITS5-1737F (5'-GGAAGTAAAAGTCGTAACAAGG-3') and ITS2-2043R (5'-GCTGCGTTCTTCATCGATGC-3') (Zhao et al., 2014). All reaction tubes contained 15 μl of Phusion^®^High-Fidelity PCR Master Mix (New England Biolabs) with 2 μM of forward and reverse primers and 10 ng of template DNA. The thermal cycler was set at 98°C for 1 min for initial denaturation, thenceforth at 98°C for 10 s (30 cycles of denaturation), at 50°C (30 s) for annealing, at 72°C (30 s) for elongation, and finally at 72°C (5 min). Thereafter, the resultant products were purified using Qiagen Gel Extraction Kit (Qiagen, Germany). With amplified products, the library was constructed and sequenced on an Illumina NovaSeq platform, and then 250 bp paired-end reads were generated.

The script from the Novogene platform was used for clipping barcode and primer sequences by Cutadapt (V1.9.12). From each sample, the operational taxonomic units (OTUs) were determined by UPARSE (Edgar, 2013), and additional biological data were also obtained. Furthermore, the representative OTU sequences were assigned species annotations. Unit (V.7.24) assisted in counting the community composition of each sample, then calculations were made for the OTUs matrix. For full-sample similarity comparison and to analyze the alpha-beta-diversity and calculate the observed species, Chao1, Shannon, Simpson, ACE, as well as Good's-coverage indices, QIIME (V.1.9.1) was employed. R software (V.2.15.3) was utilized to illustrate the rank abundance curve, dilution curve, and species accumulation box. Using Muscle (V.3.8.31), sequence alignments for ITS locus were carried out (Edgar, 2004). Moreover, FastTree 2 (V.1.9.1) software and the maximum likelihood (ML) method was chosen for phylogenetic analysis (Price et al., 2010). PCA was displayed by WGCNA packages and ggplot2 package in R software (V.1.9.1). Analysis of fungal trophic mode was conducted using the FUNGuild database (Nguyen et al., 2016), and bacterial OTUs were put into PICRUSt to predict functional analysis (Langille et al., 2013).

### Biolog Inoculation and Analysis

The BIOLOG method was employed to inspect the functional metabolic fingerprints of the community and the patterns of carbon source utilization (Classen et al., 2003). Fifty ml of 0.85% NaCl (27 mL, w/v) was mixed with 3 g of tobacco leaves (cut into pieces) and shaken for 1 h at 28°C. The mixture was then diluted at a ratio of 1:1000. Into each of the ECO micro-plate well, 150 μL of dilution was added using an electronic pipette and incubated at 28°C in darkness for 7 days. The Biolog OmniLog^®^ system (BIOLOG, Hayward, CA, USA) used in this study was equipped with a charge-coupled device camera system and an incubator. The color level of each well was measured in omnilog units (OUs) every 30 min by OmniLog digital camera. The value of OU is the reflection of the dynamic development of microbe carbon metabolism, represented by the utilization of carbon metabolism. OU and optical density (OD_620nm_) are comparable and could be calculated as 500 times the OD (Cruz et al., 2021). Heatmap illustrator (HemI 1.0.3.3) was used to display the heatmap of the maximum OU values of 16 samples.

### Data and Statistical Analysis

IBM SPSS Statistics 23 (IBM Corp., New York, USA) was used to analyze the data and compare the differences in alpha-diversity indices of fungal and bacterial communities (Mao et al., 2020). A multiple comparison was performed by the LSD test (P < 0.05) to compare the diversity index of different treatments. The mean values were compared and the *P* ≤ 0.05 was considered to be statistically significant.

## Results

### Diversity and Abundance of Phyllospheric-Culturable Microorganism

By tissue isolation method, 153 fungal isolates belonging to 33 genera were obtained, while plate coating yielded 99 bacterial isolates belonging to 15 genera (Supplementary Table 2). For fungi, more genera were isolated on PDA (25) than AEA (24). The most predominant genus was *Cercophora* comprising 9 of 75 isolates on PDA and 14 of 78 on AEA, followed by *Coprinopsis* with 8 isolates on PDA and 13 isolates on AEA. The third abundant genus was *Alternaria* with 16 isolates (8 isolates each on PDA and AEA, respectively). The rarest genera were *Brunnipila, Coniochaeta, Gibellulopsis, Hypoxylon, Irpex, Peniophora, Preussia, Myrmaecium, Rhizopus, Zopfiella, Schizophyllum*, and *Verticillium* with 1 isolate on either PDA or AEA. The number of genera at three collection stages were 13, 17, and 25, respectively, inferring that the fungal diversity gets richer as the tobacco leaves become mature. For bacteria, more genera were isolated on LB (15) than on NA (13). The most abundant genus was *Pantoea* accounting for up to 33.33% of the bacterial isolates (17 of 53 isolates on LB and 16 of 46 isolates on NA), followed by *Pseudomonas* with 26 isolates (13 isolates on LB and on NA, respectively). The rarest genus was *Agrobacterium* with only 1 isolate on LB.

When analyzed the collection period of samples combining healthy and diseased leaves, diversity was the highest at the third sampling stage with 26 fungal and 13 bacterial genera, followed by the first and second time (13 fungal and 10 bacterial genera, 17 fungal and 4 bacterial genera, respectively). The relative abundance (percent of isolates of a genus out of the total number of isolates) at the first time showed that *Cercophora* and *Pseudomonas* were the most abundant (37.93% of fungal isolates and 29.17% of bacterial isolates), at the second time showed that *Alternaria* (19.64% of fungal isolates) and *Pantoea* (47.83% of bacterial isolates) were the most abundant, and at the third time showed that *Coprinopsis* (19.12% of fungal isolates) and *Pantoea* (42.31% of bacterial isolates) were the most abundant.

When analyzed for leaf type, by combining samples from all collection dates as well as different leaf positions, fungal diversity was higher for diseased leaves (25 genera) than healthy leaves (23 genera). In contrast, bacterial diversity for diseased leaves (10 genera) was lower than for healthy leaves (13 genera). The relative abundance in healthy groups suggested that *Coprinopsis* (18.57% of fungal isolates) and *Pseudomonas* (44.90% of bacterial isolates) were the most abundant genera. For diseased groups, the most abundant genera were *Cercophora* (15.66% of fungal isolates) and *Pantoea* (58.00% of bacterial isolates).

A comparison of microbial abundance among positions exhibited that the numbers of fungal genera from three positions (up, middle, and lower) were 16, 18, and 22, respectively. In the case of bacteria, the numbers of genera from three positions (up, middle, and lower) were 14, 5, and 9, respectively. Combining leaf position and leaf type, the number of fungal genera from the upper and lower diseased leaves (13 and 17 genera) was much higher than healthy leaves (8 and 14 genera), whereas the number of bacterial genera from the upper diseased leaves (7 genera) was less than the healthy leaves (10 genera).

### Diversity of Microbial Communities From High-Throughput Sequencing

#### Quality of Fungal and Bacterial Sequence Data

There was a total of 1,150,015 effective tags and 305,236,114 bases of fungi across 9 healthy samples and 7 diseased samples and the average length was 266 nt. A total of 1,321 OTUs of fungi at ≥ 97% nt identity were obtained after removing low-quality, chimeric, and rare sequences from 16 samples, resulting in an average of 71,876 tags per sample (Table 1). The fungal sequence of each sample was deposited in the SRA database under the accession # PRJNA751254. With regard to bacteria, after quality filtering and de-noising, the removal of mitochondrial, chloroplast DNA, and other non-bacterial sequences, a sum of 1,317,757 effective tags were classified into 358 OTUs at ≥ 97% nt identity, and 537,085,996 bases were retained from 16 samples (Table 1). The sequences of 46 samples were deposited in the SRA database under project accession # PRJNA751261. Once the fungal and bacterial sample numbers reached ~45, the species accumulation boxplot indicated that the plateau phase has been approached (Supplementary Figure 2), pertaining to the sufficiency of sampling efforts for the estimation of species richness and describing the fungal and bacterial diversity. With the increment of the sequencing, the rarefaction curves for all samples tended to level off (Supplementary Figure 3), representing that the fungal and bacterial composition had been accurately described by the sequence coverage.

**Table 1:** Data quality control of sequencing samples.

**Experimental group**	**Effective tags number**		**Total base number (nt)**		**Average length (nt)**	
	**Fungi**	**Bacterial**	**Fungi**	**Bacterial**	**Fungi**	**Bacterial**
BJY871U	61,629	84,762	17,075,800	34,406,204	278	406
BJY871M	67,542	79,846	18,274,355	32,407,702	271	406
BJY871D	73,690	77,763	19,978,818	31,564,583	271	406
BJB871D	70,448	79,824	18,899,888	32,398,620	269	406
BJY872U	72,882	82,781	19,758,827	33,598,589	271	406
BJY872M	74,436	83,113	20,076,593	33,737,812	270	406
BJY872D	72,464	82,900	19,689,731	33,647,372	272	406
BJB872U	74,021	79,602	19,706,824	32,315,498	267	406
BJB872M	75,147	82,446	20,344,614	33,463,686	271	406
BJB872D	74,070	85,497	18,881,324	35,230,959	256	412
BJY873U	62,568	84,586	17,013,952	34,342,249	272	406
BJY873M	67,094	85,755	18,097,642	34,846,101	270	406
BJY873D	71,198	91,210	19,258,046	37,028,179	271	406
BJB873U	73,307	80,302	18,884,437	33,136,672	259	413
BJB873M	78,550	84,490	19,677,941	34,660,996	251	410
BJB873D	80,969	72,880	19,617,322	30,300,776	243	416

#### Fungal and Bacterial Diversity

A comparison among sample collection periods unveiled a progressive increase in the count of fungal OTUs with time (Table 2). For fungi, the number of OTUs of diseased leaves increased significantly from the first collection time to the third time (48, 202, and 337, respectively). For bacteria, the number of OTUs of healthy leaves decreased, with a significant difference between the second and third time, while it increased from the first to the second sampling stage. For fungi, irrespective of position, healthy leaves displayed the highest diversity of OTUs contingent on the mean values of Shannon, Simpson, and Chao1 at three collection stages (Table 2). However, the diversity on diseased leaves was lower, established on the mean Shannon, Simpson, and Chao1 values in the second and third periods. The trend remained the same for the bacterial diversity of OTUs, where the lowermost diversity index values were obtained for OTUs of bacteria for diseased leaves at three collection periods.

**Table 2:** Alpha diversity index of the ITS and 16S rRNA libraries obtained for clustering at 97% identity (OTU level).

**Samples**		**fungi**					**bacteria**				
		**Shannon**	**Simpson**	**Chao1**	**Coverage**	**OTU**	**Shannon**	**Simpson**	**Chao1**	**Coverage**	**OTU**
Healthy group	BJY871U	3.07 ± 0.03a	0.80 ± 0.00a	38.14 ± 5.22c	1	47	2.79 ± 0.69abc	0.83 ± 0.09ab	10.92 ± 5.14cd	0.99	25
	BJY871M	3.14 ± 0.20a	0.79 ± 0.01a	96.18 ± 99.9a	1	197	2.93 ± 0.31ab	0.83 ± 0.06ab	27.00 ± 18.084ab	0.99	21
	BJY871D	3.04 ± 0.02a	0.79 ± 0.00a	47.44 ± 0.77bc	1	59	3.49 ± 0.32a	0.90 ± 0.03a	30.08 ± 14.32a	0.99	32
	BJY872U	3.11 ± 0.05a	0.80 ± 0.01a	48.07 ± 1.85bc	1	54	2.90 ± 0.54ab	0.82 ± 0.01ab	15.89 ± 7.92bcd	0.99	21
	BJY872M	3.15 ± 0.10a	0.80 ± 0.01a	53.23 ± 5.51abc	1	67	3.12 ± 0.34ab	0.86 ± 0.04a	17.03 ± 0.55abcd	0.98	53
	BJY872D	3.18 ± 0.02a	0.81 ± 0.01a	65.50 ± 16.39abc	1	73	2.59 ± 0.23abcd	0.78 ± 0.04ab	11.86 ± 4.40cd	0.99	15
	BJY873U	3.19 ± 0.08a	0.81 ± 0.01a	61.62 ± 8.95abc	1	80	1.90 ± 0.54cd	0.64 ± 0.19bc	7.50 ± 3.12cd	0.99	14
	BJY873M	3.17 ± 0.07a	0.80 ± 0.01a	58.30 ± 6.26abc	1	71	1.83 ± 0.46cd	0.65 ± 0.08bc	6.33 ± 3.21d	0.99	16
	BJY873D	3.19 ± 0.13a	0.80 ± 0.01a	68.45 ± 21.53bcd	1	86	2.62 ± 0.43abcd	0.79 ± 0.07ab	13.00 ± 5.68cd	0.99	23
Diseased group	BJB871D	3.14 ± 0.01a	0.79 ± 0.01a	43.47 ± 3.93c	1	48	3.49 ± 0.25a	0.90 ± 0.02a	20.17 ± 7.07abc	0.99	29
	BJB872U	3.20 ± 0.08a	0.81 ± 0.02a	58.50 ± 3.03abc	1	66	2.71 ± 0.46abc	0.82 ± 0.07ab	11.83 ± 3.75cd	0.99	15
	BJB872M	3.14 ± 0.02a	0.80 ± 0.00a	52.28 ± 1.51abc	1	65	2.77 ± 1.26abc	0.77 ± 0.24ab	13.40 ± 7.27cd	0.99	15
	BJB872D	2.19 ± 1.60b	0.56 ± 0.43b	52.67 ± 3.33abc	1	71	1.65 ± 0.85d	0.52 ± 0.26c	11.00 ± 6.56cd	0.99	16
	BJB873U	3.14 ± 0.25a	0.77 ± 0.07ab	93.74 ± 22.81a	1	115	2.53 ± 0.98abcd	0.77 ± 0.16ab	14.83 ± 11.09bcd	0.97	26
	BJB873M	3.01 ± 0.36ab	0.75 ± 0.09ab	80.43 ± 8.56abc	1	102	2.69 ± 0.39abc	0.79 ± 0.08ab	12.58 ± 3.64cd	0.98	20
	BJB873D	2.99 ± 1.01b	0.73 ± 0.23ab	91.18 ± 10.99ab	1	120	2.35 ± 0.37bcd	0.73 ± 0.10abc	9.83 ± 4.91d	0.99	17

*The data in the table are mean ± SD (n = 3). Different lowercase letters in the same column indicate significant differences in treatments at 0.05 level (p < 0.05)*.

### Microbial Taxa in the Tobacco Phyllosphere

#### Fungal Taxa Composition of the OTUs

**Figure 1:**
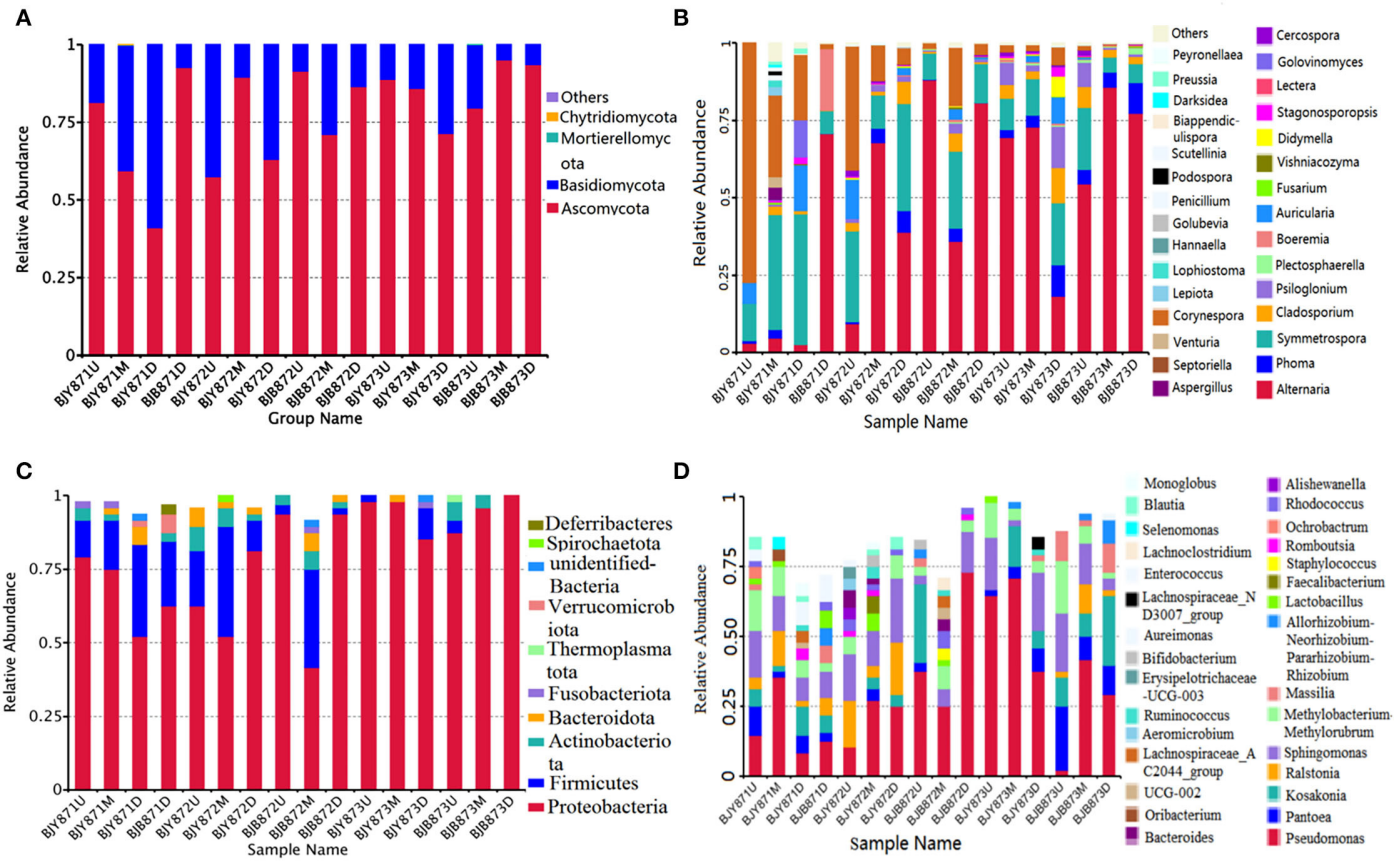
The relative abundance of 16 samples at phylum (top 10) and genus (top 30) levels. Different fungal phyla **(A)** and genus **(B)**; different bacterial phyla **(C)** and genus **(D)**. Phyla and genus making up <0.01 of total composition were classified as “Others.”

The distribution of different fungal phyla for the OTUs showed that 99.9% of the effective tags could be categorized under Ascomycota, Basidiomycota, Mortierellomycota, and Chytridiomycota. The ones unassigned were the members of other phyla and were highly unlikely to be true fungi (Figure 1A). In all healthy and diseased tobacco leaves, Ascomycota (78.02%) predominated the fungal communities and, to a lesser extent, Basidiomycota (21.97%), while Mortierellomycota and Chytridiomycota comprised <0.01% of the tags. Combining position, collection time, and different leaves, the average number of tags per time for the Ascomycota was 68.68, 76.54, and 85.74% in the three collection periods, respectively, indicating an increase as the tobacco leaves get older.

At the phylum level, the abundance of Ascomycota and Basidiomycota from healthy samples at the same position was increased during three collection stages, regardless of leaf health status, which suggests that the fungal abundance increased with the aging of tobacco leaves (Table 3). Statistical analysis revealed that there was no significant difference in the relative abundance of Ascomycota among the healthy samples with regard to different collecting stages and stalk positions; so did Basidiomycota, indicating that time-points and leaf positions could not affect the fungal community composition in the healthy leaves. For the diseased group, the relative abundance of Ascomycota from the same position was increased, and there was a significant difference between the abundance of Ascomycota in aspects of BJB871D (3.94%) and BJB873D (41.53), BJB872M (0.37%) and BJB873M (39.77%), portending that the collecting time had a significant effect on the abundance of Ascomycota in the diseased group. The abundance of Basidiomycota from 6 diseased samples had no difference except for BJB873D. A comparison of different positions at each sampling stage portended a higher relative abundance of Ascomycota from middle healthy leaves than healthy leaves from the upper and lower positions in three stages, whereas the abundance of Basidiomycota from lower healthy leaves was higher than leaves from the upper and middle healthy leaves. For diseased leaves, the abundance of Ascomycota was higher in the lower leaves than in the upper and middle leaves. While comparing two kinds of leaves from the same position, the relative abundance of Ascomycota and Basidiomycota from diseased leaves were higher than healthy leaves in three stages at three positions and the difference got more significant during three collecting stages, indicating that the time-point was a factor which could affect the fungal community composition.

**Table 3:** Community compositions and relative abundance of phyllosphere microorganism from tobacco leaves infected by *Alternaria alternata* at fungal phyla and genera levels (%).

**Leaf type**	**Collecting time**	**Leaf position**	**Sample name**	**Phyla**						**Genera**					
				**Ascomycota**	**Basidiomycota**	***Alternaria***	***Phoma***	***Symmetrospora***	***Cladosporium***	***Psiloglonium***	***Plectosphaerella***	***Boeremia***	***Auricularia***	***Fusarium***	***Vishniacozyma***
Healthy group	First time	Up	BJY871U	0.05 ± 0.01b	0.02 ± 0.02b	0.00 ± 0.00c	0.00 ± 0.00b	0.01 ± 0.01b	0.00 ± 0.00b	0.00 ± 0.00b	0.00 ± 0.00b	0.00 ± 0.00b	0.00 ± 0.00b	0.00 ± 0.00b	0.00 ± 0.00b
		Middle	BJY871M	0.43 ± 0.67b	0.09 ± 0.06b	0.03 ± 0.04c	0.01 ± 0.01b	0.04 ± 0.02b	0.02 ± 0.03b	0.00 ± 0.01b	0.00 ± 0.00b	0.00 ± 0.00b	0.00 ± 0.00b	0.00 ± 0.00b	0.00 ± 0.00b
		Lower	BJY871D	0.08 ± 0.05b	0.12 ± 0.07b	0.00 ± 0.00c	0.00 ± 0.00b	0.10 ± 0.09b	0.00 ± 0.00b	0.00 ± 0.00b	0.00 ± 0.00b	0.00 ± 0.00b	0.01 ± 0.02b	0.00 ± 0.00b	0.00 ± 0.00b
	Second time	Up	BJY872U	0.08 ± 0.05b	0.07 ± 0.05b	0.02 ± 0.01c	0.00 ± 0.00b	0.05 ± 0.04b	0.01 ± 0.01b	0.00 ± 0.00b	0.00 ± 0.00b	0.00 ± 0.00b	0.02 ± 0.03b	0.00 ± 0.00b	0.00 ± 0.00b
		Middle	BJY872M	1.68 ± 1.78b	0.08 ± 0.05b	0.02 ± 0.01c	0.04 ± 0.03b	0.08 ± 0.05b	0.02 ± 0.02b	0.03 ± 0.05b	0.00 ± 0.00b	0.00 ± 0.00b	0.00 ± 0.00b	0.00 ± 0.00b	0.00 ± 0.00b
		Lower	BJY872D	0.53 ± 0.38b	0.25 ± 0.07b	0.34 ± 0.32c	0.06 ± 0.05b	0.23 ± 0.04b	0.04 ± 0.03b	0.01 ± 0.01b	0.00 ± 0.00b	0.00 ± 0.00b	0.02 ± 0.02b	0.00 ± 0.00b	0.00 ± 0.00b
	Third time	Up	BJY873U	1.78 ± 1.04b	0.16 ± 0.12b	1.51 ± 1.11bc	0.05 ± 0.03b	0.14 ± 0.11b	0.06 ± 0.02b	0.07 ± 0.07b	0.00 ± 0.00b	0.01 ± 0.01b	0.00 ± 0.00b	0.00 ± 0.00b	0.00 ± 0.00b
		Middle	BJY873M	1.78 ± 1.70b	0.21 ± 0.11b	1.61 ± 1.73bc	0.05 ± 0.04b	0.18 ± 0.09b	0.03 ± 0.02b	0.03 ± 0.01b	0.01 ± 0.01b	0.00 ± 0.00b	0.02 ± 0.04b	0.00 ± 0.00b	0.00 ± 0.00b
		Lower	BJY873D	0.86 ± 0.65b	0.29 ± 0.36b	0.19 ± 0.19c	0.12 ± 0.18b	0.15 ± 0.13b	0.11 ± 0.11b	0.15 ± 0.20b	0.01 ± 0.01b	0.00 ± 0.00b	0.14 ± 0.23a	0.00 ± 0.00b	0.00 ± 0.00b
Diseased group	First time	Lower	BJB871D	3.94 ± 1.31b	0.23 ± 0.12b	3.47 ± 1.88bc	0.00 ± 0.00b	0.07 ± 0.10b	0.01 ± 0.00b	0.00 ± 0.00b	0.00 ± 0.00b	0.40 ± 0.56a	0.00 ± 0.00b	0.00 ± 0.00b	0.00 ± 0.00b
	Second time	Up	BJB872U	11.31 ± 8.28ab	0.33 ± 0.05b	6.39 ± 8.5abc	0.02 ± 0.02b	0.34 ± 0.06b	0.03 ± 0.01b	0.01 ± 0.00b	0.01 ± 0.01b	0.00 ± 0.00b	0.00 ± 0.00b	0.01 ± 0.00b	0.00 ± 0.00b
		Middle	BJB872M	0.37 ± 0.36b	0.12 ± 0.07b	0.25 ± 0.37c	0.01 ± 0.02b	0.09 ± 0.05b	0.02 ± 0.01b	0.01 ± 0.01b	0.00 ± 0.00b	0.00 ± 0.01b	0.02 ± 0.02b	0.00 ± 0.00b	0.00 ± 0.00b
		Lower	BJB872D	32.85 ± 55.52ab	0.17 ± 0.04b	32.69 ± 55.41ab	0.02 ± 0.03b	0.16 ± 0.04b	0.04 ± 0.05b	0.03 ± 0.04b	0.00 ± 0.01b	0.01 ± 0.01b	0.01 ± 0.01b	0.01 ± 0.01b	0.01 ± 0.01b
	Third time	Up	BJB873U	21.16 ± 33.88ab	0.76 ± 0.43b	18.31 ± 30.31abc	0.61 ± 0.83b	0.73 ± 0.40b	0.57 ± 0.61ab	0.95 ± 1.30a	0.33 ± 0.56b	0.01 ± 0.01b	0.02 ± 0.03b	0.02 ± 0.02b	0.01 ± 0.00b
		Middle	BJB873M	39.77 ± 30.17a	0.61 ± 026b	35.31 ± 26.50a	2.66 ± 2.26a	0.58 ± 0.28b	1.15 ± 1.60a	0.17 ± 0.08b	0.12 ± 0.12b	0.06 ± 0.06b	0.00 ± 0.00b	0.00 ± 0.00b	0.00 ± 0.00b
		Lower	BJB873D	41.53 ± 32.62a	2.27 ± 1.79a	36.33 ± 32.30a	2.44 ± 2.82a	2.09 ± 1.62a	1.03 ± 0.84a	0.32 ± 0.24b	0.70 ± 0.65a	0.06 ± 0.04b	0.01 ± 0.02b	0.14 ± 0.21a	0.11 ± 0.17a

*The data in the table are mean ± SD (n = 3). Different lowercase letters in the same column indicate significant differences in treatments at 0.05 level (p < 0.05)*.

**Figure 2:**
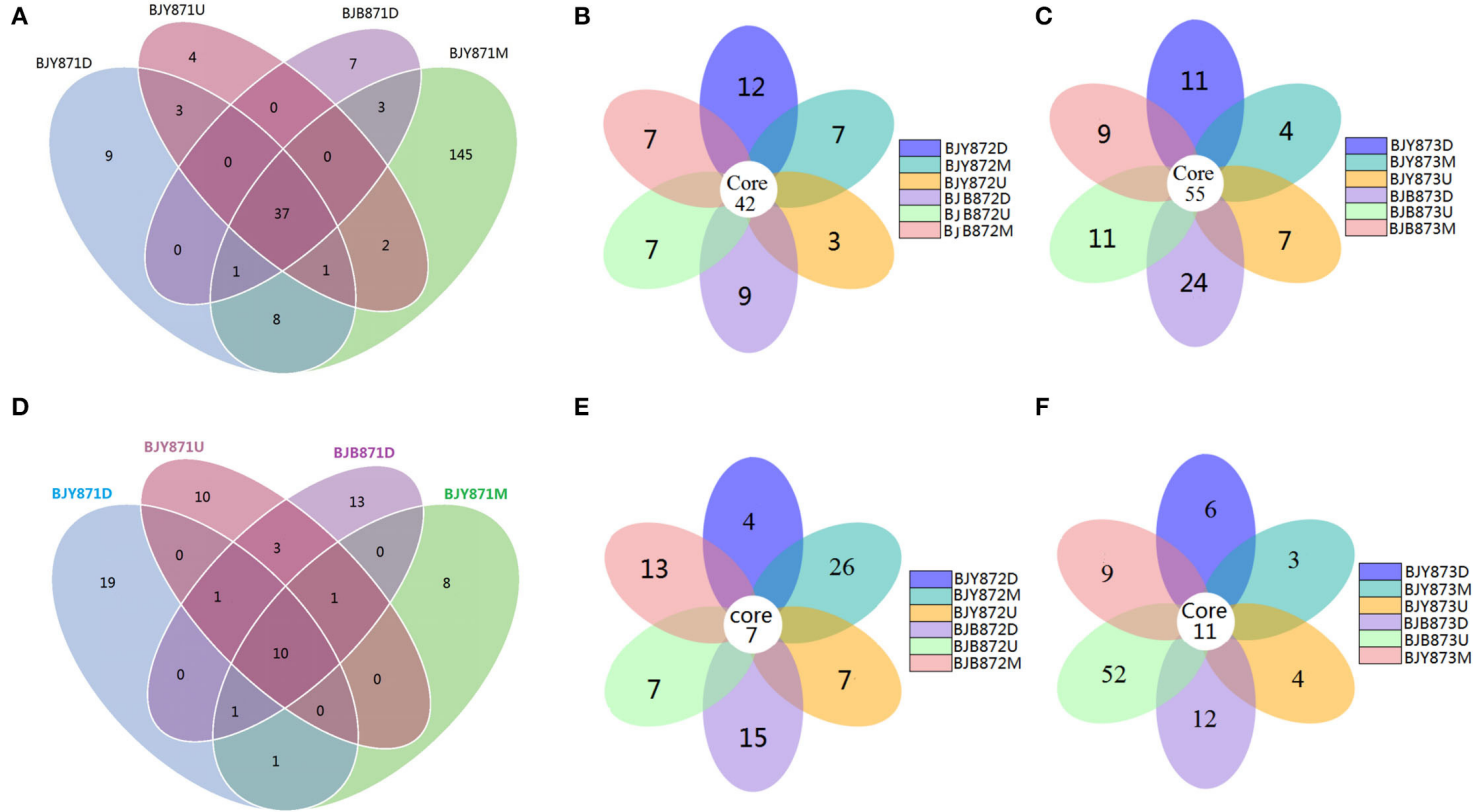
Venn diagram showing the number of operational taxonomic units (OTUs) detected in healthy and diseased tobacco leaves. Fungal Venn diagram in healthy and diseased groups from three positions **(A–C)**. Bacterial Venn diagram in healthy and diseased groups from three positions **(D–F)**.

The differences among the communities at the OTU level from healthy and diseased samples at three collection periods are depicted in Venn diagrams (Figures 2A–C). Overall, 1,321 OTUs were acquired, where 734 belonged to the healthy and 587 to the diseased group (Table 2). There were 351 OTUs discovered for the first time, and 10.54% of them were shared OTUs. Healthy leaves from the middle position contained more fungal varieties (197 OTUs). A total of 396 OTUs were obtained for the second time, and 10.61% of them were shared OTUs. In the third stage, a total of 574 OTUs were discovered, and 9.58% of them were shared OTUs. However, the number of eukaryote OTUs in the healthy group was more in contrast to the diseased group, indicating that there was a significant difference among their fungal varieties. Of note, the number of OTUs in the healthy and diseased groups increased markedly at the second and third collection time. Apropos the second collection time, 194 OTUs were obtained from the healthy leaves, and 23.2% of them were shared OTUs; 202 OTUs were discovered from the diseased leaves, and 25.74% of them were shared OTUs. Diseased leaves from the upper position contained more fungal varieties than the healthy leaves (66 and 54 OTU, respectively). Concerning the third period, 237 OTUs were discovered from healthy leaves, and 23.63% of them were shared OTUs, while there were 337 OTUs in the diseased group, and 24.93% of them were shared. Samples from the diseased leaves (upper, middle, and lower positions) featured more fungal varieties (115, 102, and 120 OTUs, respectively) than the samples from the healthy leaves (80, 71, and 86 OTUs, respectively), as presented in Supplementary Figure 4.

**Figure 3:**
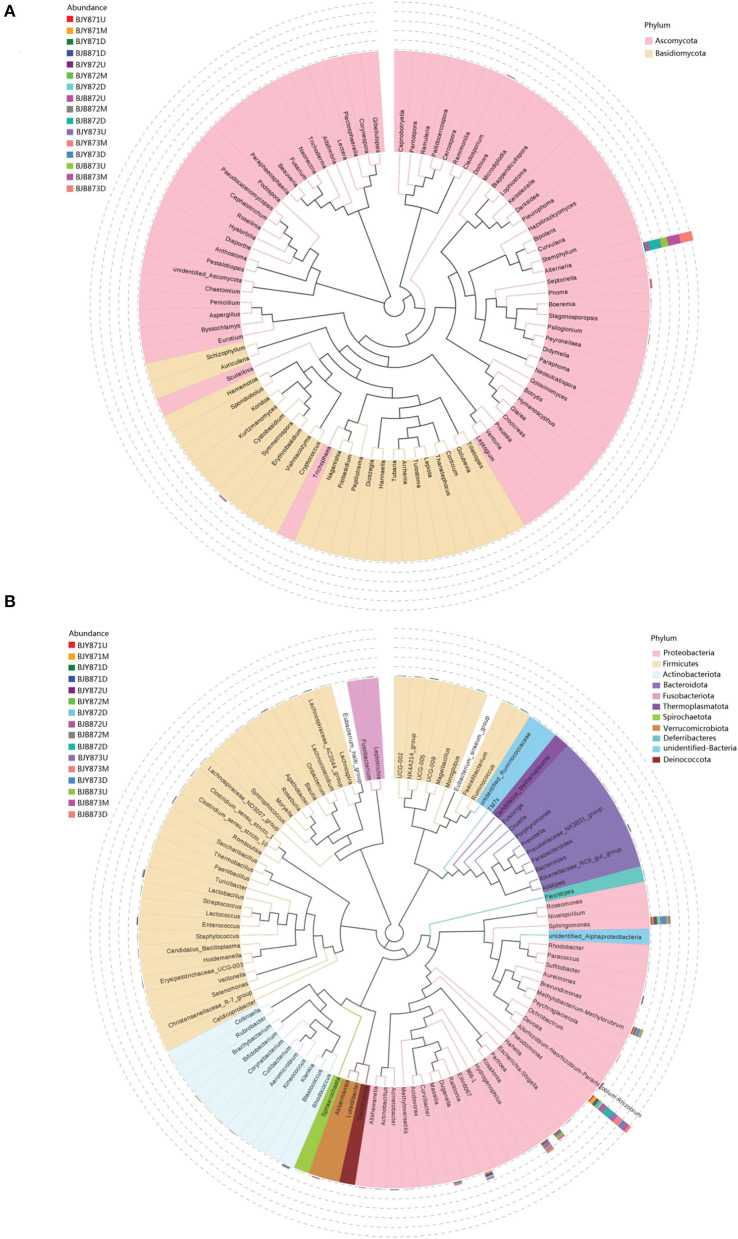
Maximum likelihood (ML) tree of 100 most abundant fungal **(A)** and bacterial **(B)** genera from the tobacco leaves infected with *Alternaria alternata* obtained by the analysis of ITS rDNA and 16S rRNA pyrosequencing data.

**Figure 4:**
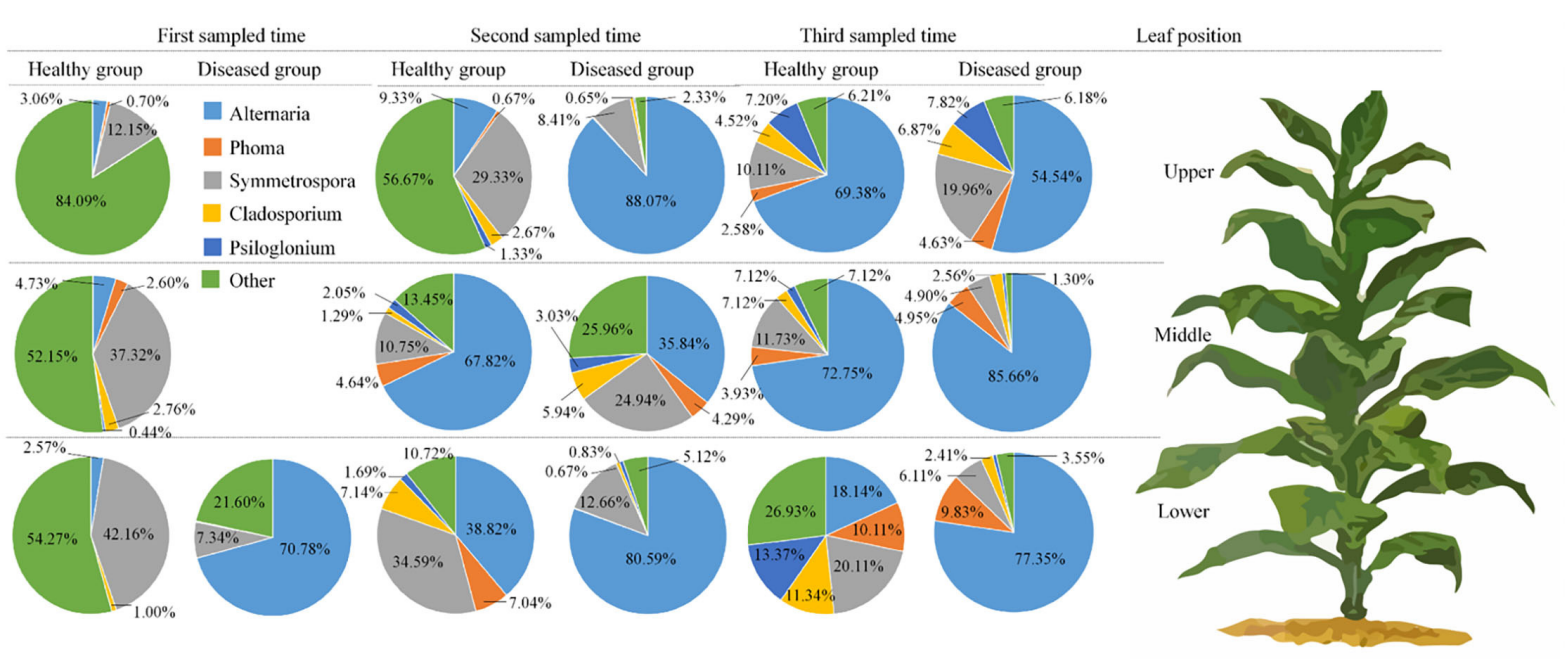
Pie charts showing the relative abundance of top 5 fungal genus of Alternaria, Phoma, Symmetrospora, Cladosporium, Psiloglonium, and “others” in the three analyzed leaf positions and at three collecting time.

From 16 samples, 87 genera of fungi were obtained, and the most frequent ones are shown in Figure 1B, and the abundance of the top 10 genera is shown in Table 3. Out of those 30 genera, the 10 highest numbers of tags were for *Alternaria, Phoma, Symmetrospora, Cladosporium, Psiloglonium, Plectosphaerella, Boeremia, Auricularia, Fusarium*, and *Vishrniacozyma*. *Alternaria*, which would include the tobacco leaf brown spot pathogen “*A. alternata*,” had 48.71% of the tags. According to the ML tree of the 100 most abundant fungal genera, the dominant fungi were associated with Ascomycota, followed by Basidiomycota (Figure 3A). On the part of Ascomycota, *Alternaria* remained the dominant genus, including *A. alternata*, followed by *Phoma, Cladosporium, Psiloglonium*, and *Boeremia* while *Symmetrospor*a was the dominant genus of Basidiomycota. The abundance of the top five genera varied substantially among the samples (Figure 4) and statistical analysis for the top ten genera was shown in Table 3. Apparently, *Alternaria* was the dominant phyllospheric fungus in 7 diseased samples and the abundance of diseased samples from the same position was increased during three collection stages. On comparing the diseased leaves from the same collecting time, the abundance of the top ten genera did not show any differences, while when regarding to time-points, there was a significant difference between the abundance of *Alternaria* in aspects of BJB871D (3.47%), BJB873D (36.33%), BJB872M (0.25%), and BJB873M (35.31%), and the results were the same regarding the abundance of *Phoma, Symmetrospora, Cladosporium, Fusarium*, and *Vishniacozyma* in the lower diseased samples. The abundance of *Phoma* from the same position in the same leaf type increased during the course of three sampling stages; moreover, *Symmetrospora* was found to be the dominant genus in healthy leaves at the first stage. Furthermore, the abundance of *Phoma, Symmetrospora*, and *Cladosporium* from the same position at the third collecting stage in two kinds of leaf types had significant differences; they were higher in diseased leaves, while they had no difference at the first and second stages, indicating that that time-point was a factor which could affect the fungal genus composition. However, the abundance of the top ten genera of the healthy group had no difference during the three stages.

#### Bacterial Taxa Composition of OTUs

The 16S data-set showed that a total of 97.72% effective tags could be classified into the Proteobacteria, Firmicutes, Actinobacteriota, Bacteroidota, Fusobacteriota, Thermoplasmatota, Verrucomicrobiota, Spirochaetota, and Deferribacteres. Members of the other phyla were unassigned or unidentified (Figure 1C). In all healthy and diseased leaves, the bacterial communities were dominated by the Proteobacteria (78.65%), followed by the Firmicutes (12.77%), Actinobacteriota (2.99%), and Bacteroidota (1.82%), while Fusobacteriota, Thermoplasmatota, Verrucomicrobiota, Spirochaetota, and Deferribacteres comprised <1.00% of the tags.

The abundance of main phyla and genera for 16 samples are shown in Table 4. It was found that the abundance of Proteobacteria in healthy leaves from the upper and middle positions decreased firstly, then increased at the third stage while the abundance of Firmicutes increased and then decreased. However, in lower healthy leaves, the abundance of Proteobacteria increased from 52.08% at the first collecting stage to 85.42% at the third stage, while the abundance of Firmicutes and Bacteroidota decreased continuously. In the diseased group, the abundance of Proteobacteria increased and Firmicutes decreased. Moreover, there was a significant difference between the abundance of Proteobacteria in aspects of BJB871D (69.92%) and BJB873D (100%), and the abundance of Firmicutes regarding BJB872M (33.33%) and BJB873M (0.00%), and Actinobacteriota had no differences among 16 samples. It was noted that the rhizobia of *Neorhizobium, Mesorhizobium, Pararhizobium, Methylorubrum*, and *Methylobacterium* were found by metagenomics (Figure 1D).

**Table 4:** Community compositions and relative abundance of phyllosphere microorganism from tobacco leaves infected by *Alternaria alternata* at bacterial phyla and genera levels (%).

**Leaf type**	**Collecting time**	**Leaf position**	**Sample name**	**Phyla**					**Genera**				
				**Proteobacteria**	**Firmicutes**	**Actinobacteriota**	**Bacteroidota**	***Pseudomonas***	***Pantoea***	***Kosakonia***	***Ralstonia***	***Sphingomonas***	***Methylobacterium-Methylorubrum***
Healthy group	First time	Up	BJY871U	79.17 ± 18.04abc	12.50 ± 10.83abc	4.17 ± 3.61a	0.00 ± 0.00b	14.58 ± 20.09de	10.42 ± 18.04ab	6.25 ± 10.83c	4.17 ± 7.22ab	16.67 ± 28.87a	14.58 ± 15.73ab
		Middle	BJY871M	75.00 ± 22.53abcd	16.67 ± 18.04abc	2.08 ± 3.61a	2.08 ± 3.61ab	35.42 ± 30.83bcde	2.08 ± 3.61b	2.08 ± 3.61c	12.50 ± 6.25ab	12.50 ± 6.25a	10.42 ± 9.55ab
		Lower	BJY871D	52.08 ± 37.67cd	31.25 ± 28.64ab	0.00 ± 0.00a	6.25 ± 0.00a	8.33 ± 7.22de	6.25 ± 10.83ab	10.42 ± 13.01bc	2.08 ± 3.61ab	8.33 ± 9.55a	6.25 ± 0.00ab
	Second time	Up	BJY872U	62.50 ± 16.54bcd	18.75 ± 27.24abc	8.33 ± 14.43a	6.25 ± 6.25a	10.42 ± 13.01de	0.00 ± 0.00b	0.00 ± 0.00c	16.67 ± 28.87ab	16.67 ± 18.04a	6.25 ± 10.83ab
		Middle	BJY872M	52.08 ± 29.54cd	37.50 ± 21.65a	6.25 ± 6.25a	2.08 ± 3.61ab	27.08 ± 25.26de	4.17 ± 7.22ab	4.17 ± 3.61c	4.17 ± 3.61ab	12.50 ± 6.25a	0.00 ± 0.00b
		Lower	BJY872D	81.25 ± 10.83abc	10.42 ± 7.22abc	2.08 ± 3.61a	2.08 ± 3.61ab	25.00 ± 22.53de	0.00 ± 0.00b	4.17 ± 7.22c	18.75 ± 22.53a	22.92 ± 13.01a	8.33 ± 9.55ab
	Third time	Up	BJY873U	97.92 ± 3.61a	2.08 ± 3.61c	0.00 ± 0.00a	0.00 ± 0.00b	64.58 ± 23.66abc	2.08 ± 3.61b	0.00 ± 0.00c	0.00 ± 0.00b	18.75 ± 16.54a	12.50 ± 10.83ab
		Middle	BJY873M	97.92 ± 3.61a	0.00 ± 0.00c	0.00 ± 0.00a	2.08 ± 3.61ab	70.83 ± 14.43ab	4.17 ± 3.61ab	14.58 ± 15.73abc	0.00 ± 0.00b	2.08 ± 3.61a	4.17 ± 3.61ab
		Lower	BJY873D	85.42 ± 25.26abc	10.42 ± 18.04abc	0.00 ± 0.00a	0.00 ± 0.00b	37.50 ± 16.54abcde	8.33 ± 14.43ab	6.25 ± 10.83c	0.00 ± 0.00b	20.83 ± 20.09a	4.17 ± 3.61ab
Diseased group	First time	Lower	BJB871D	62.92 ± 18.76bcd	21.42 ± 15.65abc	4.17 ± 3.61a	0.00 ± 0.00b	11.74 ± 6.39de	2.08 ± 3.61b	4.17 ± 7.22c	4.17 ± 7.22ab	12.50 ± 10.83a	2.08 ± 3.61b
	Second time	Up	BJB872U	91.67 ± 7.22ab	4.17 ± 3.61bc	4.17 ± 3.61a	0.00 ± 0.00b	37.75 ± 6.27abcde	3.13 ± 4.42b	29.30 ± 9.59a	0.00 ± 0.00b	3.13 ± 4.42a	3.13 ± 4.42b
		Middle	BJB872M	41.67 ± 46.07d	33.33 ± 34.42a	6.25 ± 6.25a	6.25 ± 6.25a	25.00 ± 38.02de	0.00 ± 0.00b	0.00 ± 0.00c	0.00 ± 0.00b	6.25 ± 6.25a	8.33 ± 9.55ab
		Lower	BJB872D	93.75 ± 6.25ab	2.08 ± 3.61c	2.08 ± 3.61a	2.08 ± 3.61ab	72.92 ± 19.09a	0.00 ± 0.00b	0.00 ± 0.00c	0.00 ± 0.00b	14.58 ± 9.55a	4.17 ± 3.61ab
	Third time	Up	BJB873U	87.50 ± 12.50ab	4.17 ± 3.61bc	6.25 ± 6.25a	0.00 ± 0.00b	2.08 ± 3.61e	22.92 ± 29.54a	10.42 ± 7.22bc	2.08 ± 3.61ab	20.83 ± 21.95a	18.75 ± 18.75a
		Middle	BJB873M	95.83 ± 7.22ab	0.00 ± 0.00c	4.17 ± 7.22a	0.00 ± 0.00b	41.67 ± 26.02abcd	8.33 ± 9.55ab	8.33 ± 3.61bc	10.42 ± 13.01ab	14.58 ± 7.22a	6.25 ± 6.25ab
		Lower	BJB873D	100.00 ± 0.00a	0.00 ± 0.00c	0.00 ± 0.00a	0.00 ± 0.00b	29.17 ± 34.42cde	10.42 ± 13.01ab	25.00 ± 27.24ab	2.08 ± 3.61ab	4.17 ± 3.61a	2.08 ± 3.61b

*The data in the table are mean ± SD (n = 3). Different lowercase letters in the same column indicate significant differences in treatments at 0.05 level (p < 0.05)*.

Combining position, sampling time, and different leaves, the relative abundance of OTUs per time for the Proteobacteria was 67.19, 70.83, and 94.10% in the first, second, and third times, respectively, indicating an increase as the tobacco leaf get mature. While the relative abundance of OTUs per time for the Firmicutes was 20.57, 17.53, and 2.78% in the three collection stages, respectively, indicating a significant decline as tobacco leaves get mature. For healthy and diseased leaves and in all the samples, the relative abundance of Proteobacteria and Firmicutes had the same upward/down trend. The differences between the communities at the OTU level from healthy and diseased samples at three collection periods are portrayed in Venn diagrams (Figures 2D–F). A sum of 107 OTUs was detected for the first time, and 9.35% of them were shared OTUs. Healthy leaves from the lower position exhibited more bacterial varieties (32 OTUs). In the second stage, a total of 135 OTUs were discovered from the healthy and diseased leaves with 7 shared OTUs. About 89 OTUs were obtained from the healthy leaves, and 10.36% of them were shared OTUs. Healthy leaves contained more bacterial varieties than the diseased leaves (53 and 15 OTUs, respectively), from the same position (middle position). For the third stage, a total of 53 OTUs were discovered from the healthy leaves with 26.42% of them being shared, while 63 OTUs were discovered from the diseased leaves, and 36.51% of them were shared. Samples from the healthy leaves contained more bacterial varieties (78, 89, and 53 OTUs, respectively), than the samples from the diseased leaves (29, 46, and 63 OTUs, respectively), as shown in Supplementary Figure 5.

**Figure 5:**
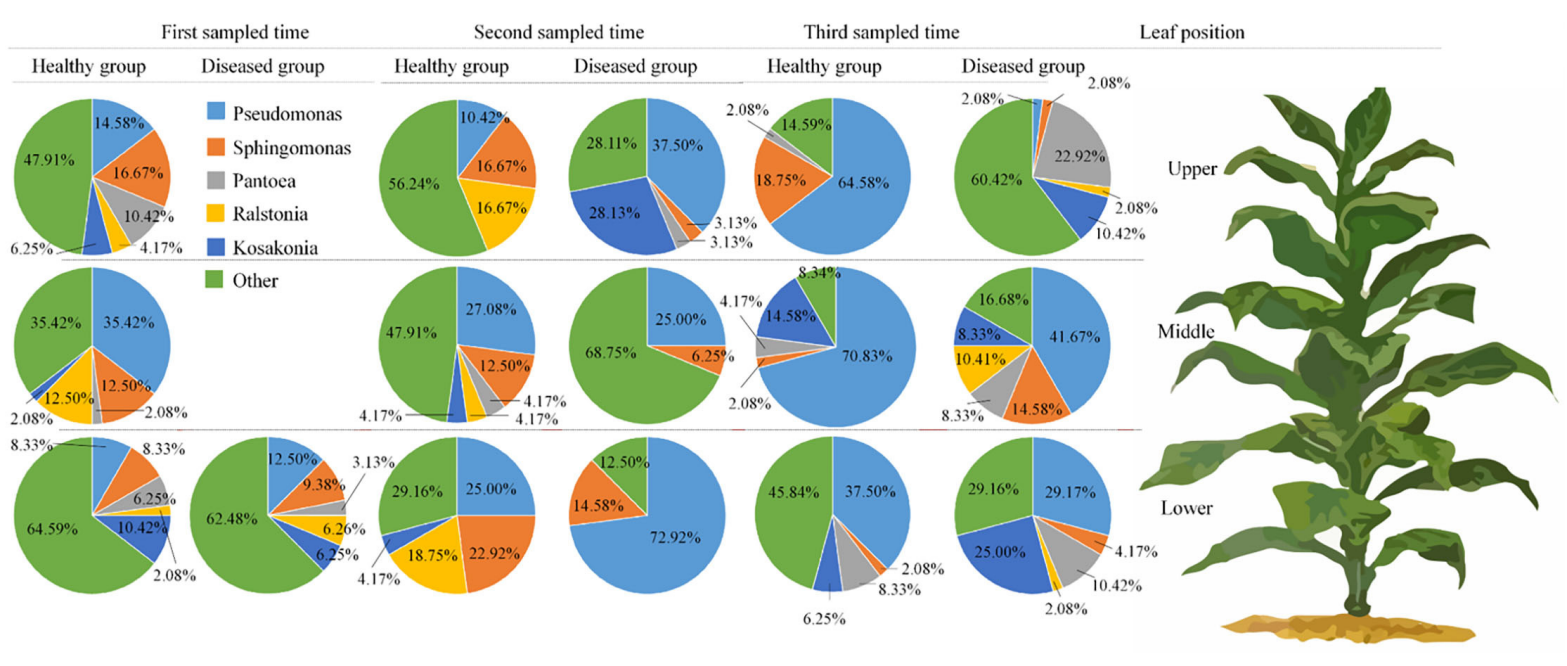
Pie charts showing the relative abundance of top 5 bacterial genus of Pseudomonas, sphingomonas, Pantoea, Ralstonia, Kosakonia, and “others” in the three analyzed leaf positions and at three collecting time.

Figure 1D represents the 30 most common bacterial genera; the top ten among those were *Pseudomonas, Pantoea, Kosakonia, Ralstonia, Sphingomonas, Methylobacterium-Methylorubrum, Faecalibacterium, Lactobacillus, Massilia*, and *Allorhizobium-Neorhizobium-Pararhizobium-Rhizobium. Pseudomonas* was the highly recurring genus in most of the healthy and diseased groups (Figure 5). There was no difference between the abundance of main genera among 16 samples based on statistical analysis (Table 4), indicating that the bacterial genera were not affected by time-point, leaf type, and stalk position. The 100 most abundant bacterial genera, according to the ML tree method, fell under the phylum of Proteobacteria, then Firmicutes, followed by Actinobacteria and Bacteroidota (Figure 3B). *Pseudomonas, Sphingomonas, Kosakonia, Methylorubrum, Pantoea*, and *Ralstonia* were the dominant genera of Proteobacteria. For Firmicutes, the dominant bacterial genera were *Lactobacillus, Enterococcus, Ruminococcus*, and *Blautia*. For the Actinobacteria and Bacteroidota, the dominant genera were *Rhodococcus* and *Bacteroides*, respectively. It is worth mentioning that *Ralstonia* was among the top ten bacterial genera and was detected in 10 samples.

### Spatial Distribution of Microbial Communities

**Figure 6:**
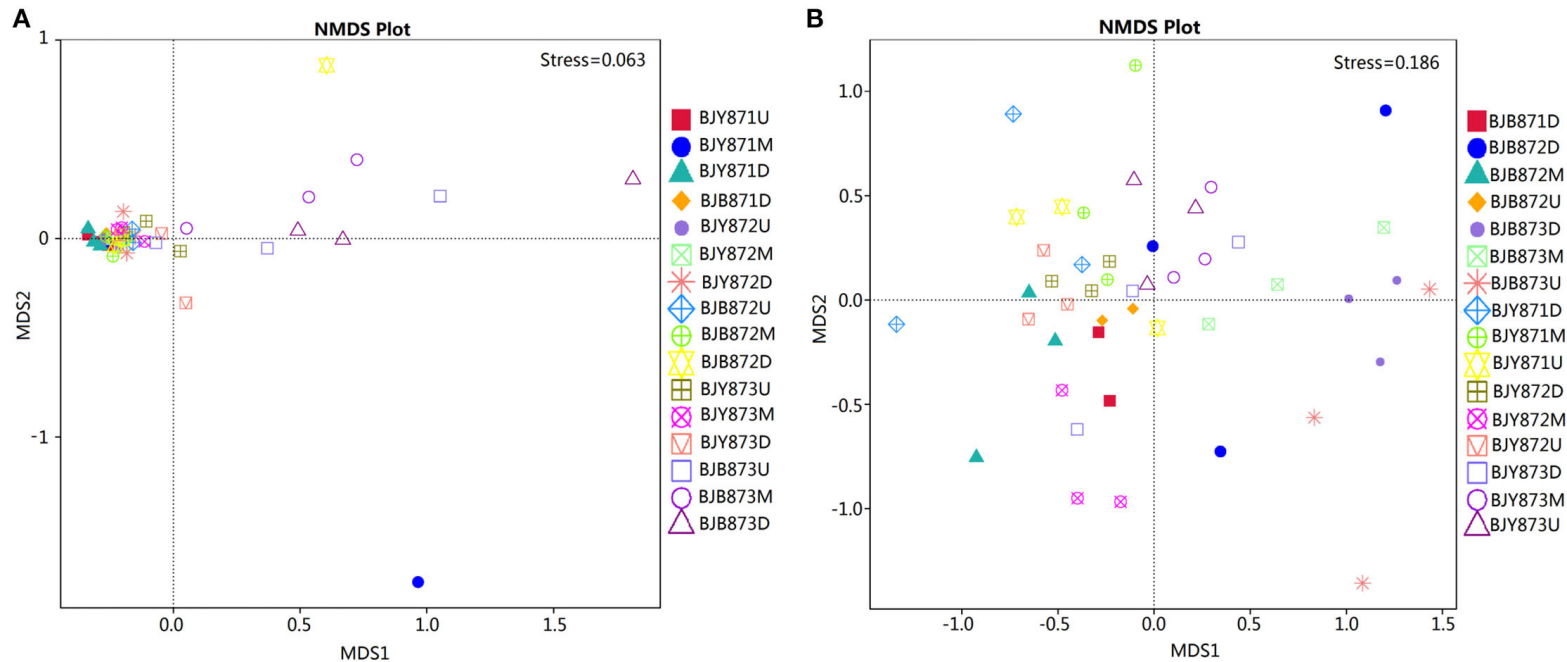
Non-metric multi-dimensional scaling (NMDS) analysis of the fungal **(A)** and bacterial **(B)** communities. The spot represents one sample, the distance between two spots represents the degree of deviation, and the same color represents samples in one group. Stress value is <0.2, indicating that NMDS could accurately reflect the difference of microbial communities among samples.

Non-metric multi-dimensional scaling (NMDS) served to evaluate the difference between samples in species complexity (Figure 6). In relation to fungal communities, all healthy samples tended to cluster together, except for three samples, which were at the middle position in the first collection stage and at the upper and lower positions in the third stage. The communities of diseased groups from the first and second stages overlapped with each other, while the communities of diseased samples collected at the third stage were evidently separated from one another (Figure 6A). Regarding bacterial communities, although all diseased and healthy groups were scattered, the samples from the same group were closer to each other, respectively (Figure 6B). The results corroborate significant differences between healthy and diseased tobacco leaves pertaining to the fungal and bacterial communities, as affected by *A. alternata*.

### Microbial Functional Prediction

**Figure 7:**
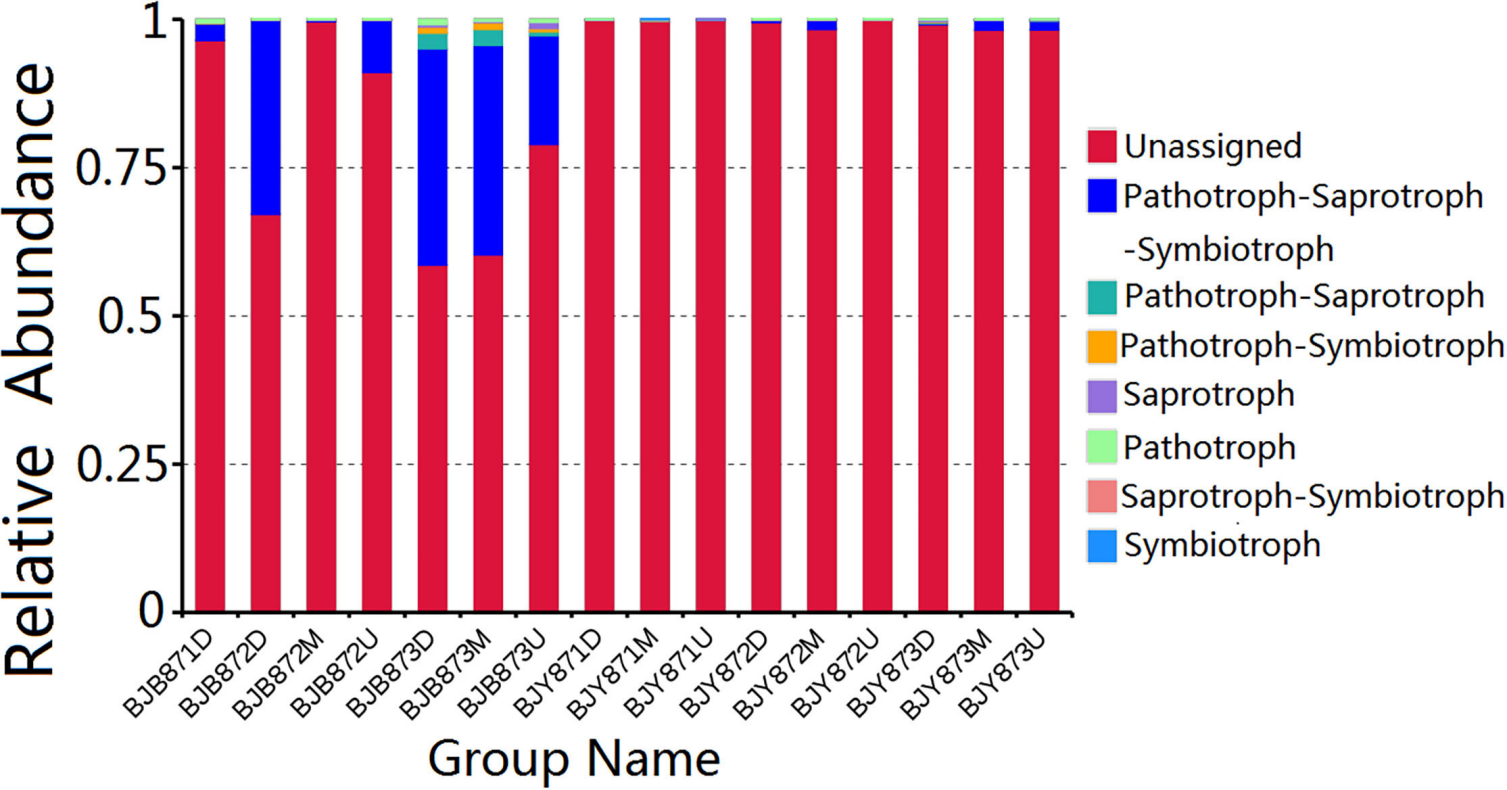
Relative abundance of fungal functional groups (guilds) contingent on OTU annotation table with disturbance frequency level.

FUNGuild database assisted in analyzing the fungal ecological functions. According to their trophic mode, the fungal OTUs were divided into 8 groups. The gene sequences in the 16 samples were mostly involved in the pathotroph-saprotroph-symbiotroph (8.88%), followed by pathotroph-saprotroph, and pathotroph-symbiotroph at 0.42 and 0.20%, respectively. However, the unassigned sequences scored 90.16% and constituted the largest group (Figure 7). Strangely, saprotroph-symbiotroph and symbiotroph only appeared in the BJY871M, in the healthy leaves collected from the middle position at the first sampling stage. In both healthy and diseased groups, the relative abundance of pathotroph-saprotroph, pathotroph-saprotroph-symbiotroph, and pathotroph-symbiotroph increased from the first collection stage to the last.

**Figure 8:**
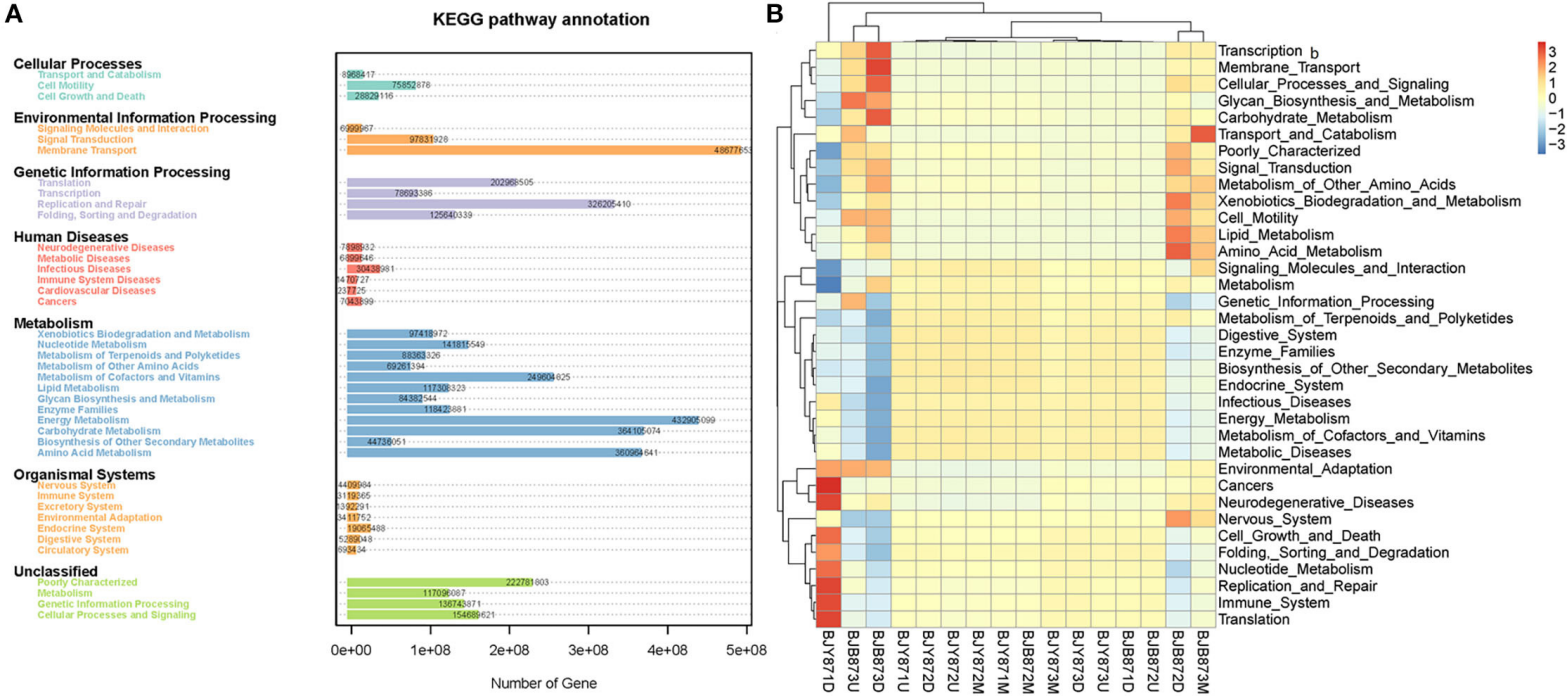
Variation of bacterial functional categories based on qualified sequences analyzed by PICRUSt. The KEGG pathway annotation of genes predictions from 16 samples **(A)**. The cluster heatmap at the KEGG level 2 function **(B)**.

Variation of bacterial functional categories was carried out by PICRUSt. There were six biological metabolism pathways based on the KEGG PATHWAY database except for the unassigned sequences (Figure 8A). The predominant pathway was metabolism function (50.03%), followed by genetic information processing (16.93%), environmental information processing (13.62%), cellular processes (2.61%), and human diseases (1.24%). At KEGG level2 function, the top thirty-five sub-functions were presented by cluster heatmap (Figure 8B). The relative abundances of the top ten sub-functions were as follows: membrane transport (11.21%), energy metabolism (10.00%), carbohydrate metabolism (8.39%), amino acid metabolism (8.34%), replication and repair (7.55%), metabolism of cofactors and vitamins (5.76%), poorly characterized (5.14%), translation (4.70%), cellular processes and signaling (3.57%), and nucleotide metabolism (3.28%). When analyzed for the position, the relative abundance of the top thirty-five sub-functions remained the same at three collection stages both in healthy and diseased groups. Similarly, when analyzed for the collection time, the relative abundance of the top thirty-five sub-functions from three positions was very similar, nullifying the likelihood of any difference among the 16 samples.

### Microbial Community Metabolic Profiles

**Figure 9:**
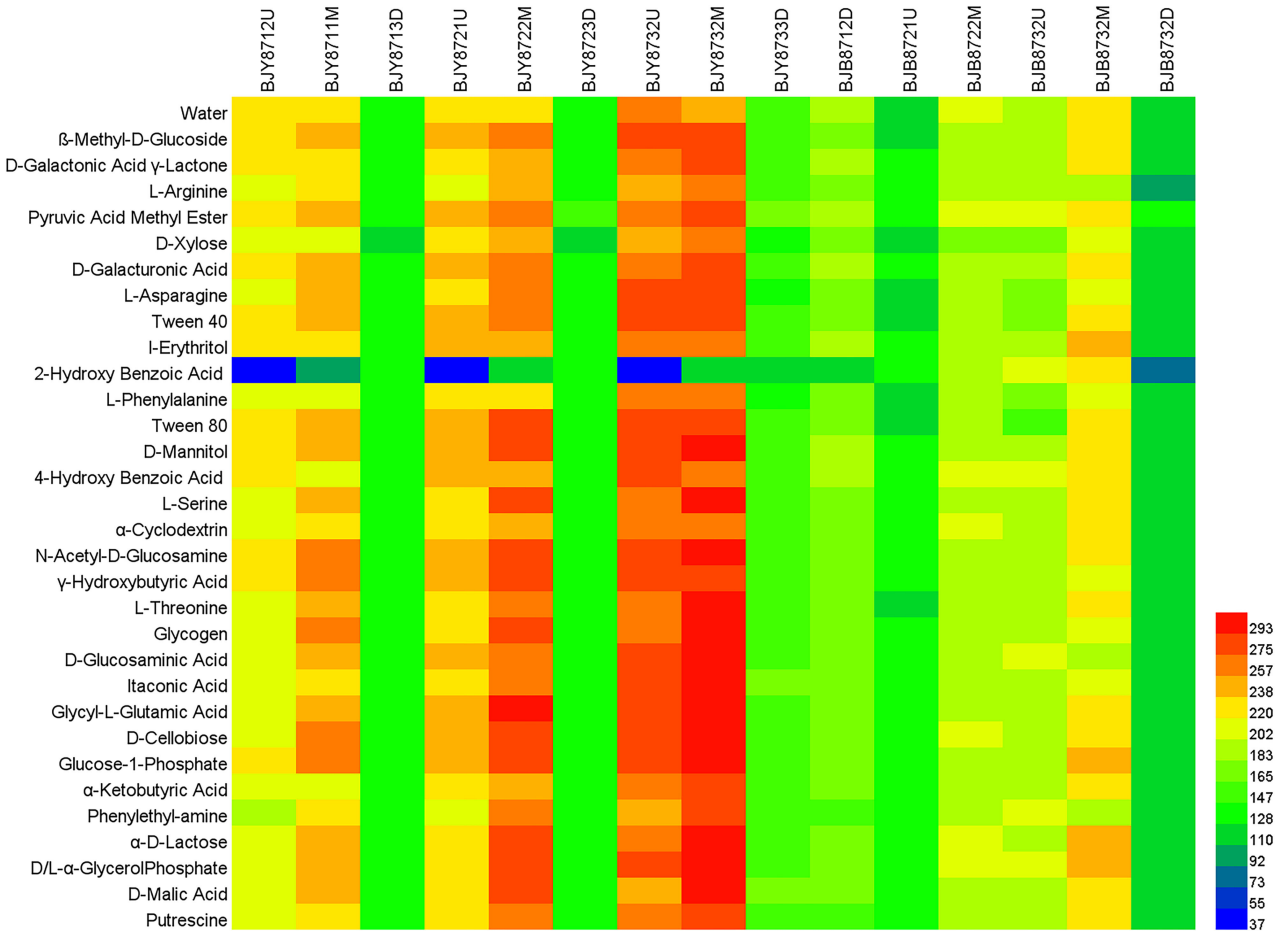
Omnilog units (OUs) of carbon sources in BIOLOG ECO-Plate of microbial communities from different samples. The maximum OU values of 16 samples are shown.

This study made use of BIOLOG ECO, which is a plate of 96 wells having 3 parallels and one blank control with 31 different carbon sources, including carboxylic acids, carbohydrates, amino acids, amines, polymers, and phenolic compounds. The maximum color changes of carbon substrates observed in the fingerprint pattern provide useful carbon source utilization profiles of microbial communities (Figure 9). Among the six carbon substrate classes, the average OU values of carbohydrates (containing D-cellobiose, D-xylose, I-erythritol, α-D-lactose, D-mannitol, N-acetyl-D-glucosamine, β-methyl-D-glucoside, Glucose-1-phosphate, D-galactonic acid γ-lactone, and D,L-α-Glycerol Phosphate) were the highest in 16 samples, implying that microbial communities had strong utilization ability of carbohydrates, followed by the carboxylic acids (including D-glucosaminic acid, D-galacturonic acid, γ-hydroxy butyric acid, pyruvic acid methyl ester, α-ketobutyric acid, D-malic acid, and itaconic acid). The polymers (Tween-40, Tween-80, α-cyclodextrin, and glycogen), amines (phenylethyl-amine and putrescine), and amino acids (including L-serine, glycyl-L-glutamic acid, L-threonine, L-arginine, L-asparagine, and L-phenylalanine) were also effectively degraded by the microbial communities. The microbial communities had the lowest OU value of phenolic compounds (2-hydroxy benzoic acid and 4-hydroxy benzoic acid), suggesting that they had the lowest utilization of phenolic compounds.

The utilization characteristics of substrates in the microbial communities of the two leaf groups remained the same. In the healthy group, the average OU values of 6 carbon sources increased from the first to the third collection stage, as in the diseased group, probably due to the changes in microbial community composition with the environmental factors. However, the average OU values of 6 carbon sources of the healthy group were higher than the diseased group except for the phenolic compounds. It signified that the microbial community of the healthy group had higher utilization rates of carbohydrates, polymers, amino acids, carboxylic acids, and amines, but lower utilization rates of phenolic compounds. Comparisons based on the leaf position indicated that the OU average values of 6 carbon sources of middle leaves were the highest, followed by the upper and lower leaf, referring to the highest utilization of 6 carbon sources by microorganisms associated with the middle leaf. The utilization of 6 carbon sources varied among 16 samples; the sample of BJY8732M had the highest OU average values. It indicated that microbes had the highest metabolism in the maturation period among the three collection stages. The microbial community on the third sampling time showed the highest carbohydrates and carboxylic acids utilization rates, with the average OU values of 289.77 and 289.40, respectively. The sample of BJB8732D had the lowest OU average values, perhaps due to fewer available nutrients for the microorganisms in a leaf getting too old.

## Discussion

In this study, three methods (culturomics, metagenomics, and BIOLOG) were employed to explore the dynamics of community diversity and functional response of phyllospheric microbes from healthy and diseased tobacco leaves infected by *A. alternata* in the spatio-temporal varying ecosystem (Rotem, 1994; Lv et al., 2013; Liu et al., 2020). More fungal genera were isolated from diseased leaves, while the bacterial genera from diseased leaves were less than healthy leaves by a traditional plating technique. Besides, microbial genera were the highest at the third sampling stage. In high-throughput sequencing technology, the dominant fungal and bacterial phyla were Ascomycota and Proteobacteria, respectively, which is consistent with the results of a previous study (Huang et al., 2021). The top five fungal genera were *Alternaria, Phoma, Symmetrospora, Cladosporium*, and *Psiloglonium*, whereas the top five bacterial genera were *Pseudomonas, Pantoea, Kosakonia, Ralstonia*, and *Sphingomonas*; the dominant fungal and bacterial genus were *Alternaria* and *Pseudomonas* which were consistent with the result of previous studies (Xu et al., 2014; Liu et al., 2019, 2020). More OTUs were obtained from the healthy group than the diseased group (Huang et al., 2020; Xiang et al., 2020). Moreover, the number of fungal OTUs was subject to change with an increase in disease severity. The α-diversity indices of fungi showed that there were no differences in the healthy group over time at the three leaf positions (Huang et al., 2021). A significant difference based on leaf types was observed where the relative abundance of predominant phyla, Ascomycota, and Basidiomycota from leaves infected by *A*. *alternata*, were found higher than healthy leaves from the same position at the three stages. *Alternaria* was the dominant genus in eleven samples except for the healthy leaves in the first stage, and at the upper and middle healthy leaves in the second stage. The relative abundance of *Phoma* from the same position in the same leaf type increased in three stages and had no significant difference among 9 healthy samples while it indicated a significant difference at 0.05 level in diseased samples at the lower and middle positions in the first and last stages. In terms of bacterial phyla and genera, Proteobacteria and *Pseudomonas* were the dominant ones in 16 samples, respectively, coinciding with Zhang's report (Zhang et al., 2020). The gene sequences in sixteen samples were mostly involved in the Pathotroph-saprotroph-symbiotroph and it was the main fungal trophic mode. The predominant pathway and sub-functions were metabolism function and membrane transport. Microbial communities had a strong utilization ability of carbohydrates with the lowest utilization of phenolic compounds.

A comparative analysis of the cultivable microbial genera counts isolated from four different media (AEA and PDA, LB, and NA) did not demonstrate any statistical difference, signifying that the media composition had no differential impact whatsoever on the detection of microorganisms. Due to complex or restricted nutritional or environmental requirements, only a minute percentage of microorganisms could be cultured in the lab (Ma et al., 2017; Li et al., 2018); concurrently, it is reported that about 0.1–3% of bacteria are readily cultivable (Wagner et al., 1993; Amann et al., 1995). In this study, 33 fungal and 15 bacterial genera were obtained by culturomics, while 87 fungal and 159 bacterial genera were found by metagenomics, showing the limitation of the culture-dependent method. The fungal genera frequently yielded by this method were *Coprinopsis, Cercophora*, and *Alternaria*, while *Coprinopsis* and *Cercophora* were non-pathogens. When comparing fungal genus counts from two kinds of leaf types in three stages, there were significant differences at the second stage; from the15 genera isolated from diseased leaves, only 8 genera isolated from healthy leaves, whereas the number of bacterial genera showed no difference. While comparing the genera between healthy and diseased groups, it was detected that *Botrytis* was isolated only from healthy leaves with a relative abundance of 14.29%, and it was also found in BJY872D, BJY873U, BJB873U, and BJB873D by a high-throughput technique. The total is well known that *Botrytis* is a causal agent of tobacco gray mold (Zhu et al., 2002). *Mucor* had a relative abundance of 12.05% in diseased leaves and only 1.43% in healthy ones. Likewise, *Pseudomonas* and *Pantoea* were the dominant ones in both healthy and diseased groups, with the relative abundance of *Pseudomonas* being 44.90 and 58.00%, and *Pantoea* being 6.00 and 10.20% in diseased and healthy groups, respectively. These observations suggest that *Mucor* and *Botrytis* could be indicators for the appearance of tobacco brown spot, while *Pseudomonas* and *Pantoea* could be indicators for the disease index of tobacco brown spot.

Referring to the high-throughput sequencing results, Ascomycota persisted to be the dominant fungal phylum followed by Basidiomycota and Chytridiomycota. This result is in line with Liu's findings (Liu et al., 2019). The taxa responded differently to the leaf senescence process; the number of OTUs in Ascomycota and Basidiomycota increased greatly during leaf senescence in the same stalk position from one leaf type, and were higher for the diseased leaves than the healthy leaves from the same position at one stage, implying that the abundance of phyla is related to disease index and is consistent with the above-mentioned conclusion that fungal OTUs was subject to change with an increase in disease severity. When healthy leaves at one collection stage were compared, the Ascomycota OTUs had a higher prevalence in the middle leaves, whereas for the lower healthy leaves, Basidiomycota had the highest OTUs scores. The data obtained with respect to bacterial phyla coincided with an earlier report that documents Proteobacteria, Firmicutes, and Actinobacteriota as the dominant bacterial phyla (Liu et al., 2020). The relative abundance of Proteobacteria remained the highest in samples from the final collection stage among 16 samples; it was higher for diseased leaves than healthy ones from the lower position in all stages. For Firmicutes, it was lower in the diseased leaves than healthy leaves from the same position at the second and third sampling periods, while they had no significant difference at 0.05 level (*p* < 0.05) in 16 samples.

At the genus level, *Alternaria, Phoma, Symmetrospora, Cladosporium*, and *Psiloglonium* were found most consistently across 16 samples, implying that they constitute the fungal “core” phyllospheric genera on tobacco. *Alternaria* was the most abundant fungal genus in thirteen samples except for three healthy samples collected at the first stage, followed by *Symmetrospora* and *Phoma*. It is well-known that tobacco is cultivated for its foliage which naturally gets mature from the bottom to the top, while this event of maturing or yellowing characterizes leaf senescence. During leaf senescence, certain macromolecules, i.e., proteins, lipids, nucleic acids, etc. are degraded and nutrients are released along with a change in the phyllospheric microbiome (Woo et al., 2013). High-throughput sequencing revealed that the abundance of *Phoma* in 16 samples from the same position increased from the first collection period to the last in both kinds of leaf types. For *Alternaria*, it increased in the upper and middle healthy leaves, while it increased from the first stage to the second and decreased at the third stage in the lower healthy and diseased leaves. Definitely, leaves from the upper and middle positions become older with the passage of time (i.e., from the first collection time to the third), whereas leaves in the lower position senesce quickly and could not provide sufficient nutrients for microbial sustenance, which very much explains the fluctuations in the growth patterns of *A. alternata*. Comparing the abundance of the same genus in the healthy group from the one in the collecting stage, the abundance of *Alternaria* from the middle position and *Symmetrospora* from the lower position was the highest among the three stalk positions. The abundance of *Alternaria* was the highest in the lower position in the diseased group, indicating that the abundance of *Alternaria* is related to the tobacco growing stage, leaf type, and leaf stalk position. There was a significant difference between the abundance of Ascomycota in aspects of BJB871D and BJB873D, BJB872M, and BJB873M; the same difference was found in *Phoma*, implying that the collecting time had a significant effect on the abundance of Ascomycota and *Phoma* in the diseased group from the lower and middle positions at 0.05 level. It should be noted that *Alternaria* and *Symmetrospora* were detected in all samples; their abundance was between 2.57 and 88.07% and 4.9 and 42.16%, respectively. The relative abundance of *Symmetrospora* was closely correlated with the abundance of *Alternaria*; when the abundance of *Alternaria* increased, the abundance of *Symmetrospora* would decrease. It is well-known that *Symmetrospora* species are red ballistosporous yeasts in the subphylum Pucciniomycotina of phylum Basidiomycota. They are ubiquitous in nature, grow as pinkish-red colonies, form nearly symmetrical ballistoconidia, and produce xanthophyll 2-hydroxytorularhodin and carotenoids (Weber et al., 2005; Almasoud et al., 2019). Understandably, *Symmetrospora* had higher relative abundances (p <0.05) in the fresh flavor style flue-cured tobacco leaves (FTLs) during the process of fermentation (Zhang et al., 2020). It is not hard to find that the abundance of “Other” from the sample at the same leaf position decreased with the age of tobacco leaves which might be related to the fact that older leaves already had lower nutrients and reduce microbial diversity.

The comparison of genera isolated by culture-dependent method and high-throughput sequencing resulted in the shared detection of eight fungal genera, and most of them were phyto-pathogens, including *Cladosporium* (Xu et al., 2014), *Didymella* (Guo et al., 2020), *Alternaria* (Wang et al., 2016), *Fusarium* (Chen et al., 2022), *Cercospora* (Kashiwa and Suzuki, 2021), *Aspergillus* (Welty and Nelson, 1971), and *Penicillium* (Pei et al., 2019). Independently, *the* culture-dependent approach showed that the top five frequently found fungal genera were *Cercophora, Coprinopsis, Alternaria, Mucor*, and *Didymella*. However, *Alternaria, Phoma, Symmetrospora, Cladosporium*, and *Psiloglonium* were particularly noticeable in the high-throughput technique. With respect to bacteria, *Pseudomonas, Pantoea*, and *Sphingomonas* were mutually detected by both techniques. In high-throughput sequencing, *Pseudomonas* and *Sphingomonas* were the commonly found bacterial genera that were noticed in all samples, with *Pseudomonas* being the most dominant one. Through the culture-dependent method, the frequently isolated bacterial genera were *Pantoea, Pseudomonas*, and *Variovorax*, whereas the *Sphingomonas* was the fifth commonly found genus. This result is in line with the findings of Liu et al. (2020), where *Pantoea* and *Pseudomonas* were declared, after high-throughput sequencing, as the dominant bacterial genera isolated from *A. alternata* infected leaves. Yet it is worth noting that rhizobia including *Neorhizobium, Mesorhizobium, Pararhizobium, Methylorubrum*, and *Methylobacterium* were found by metagenomics. This result is in line with the previous report that their presence has also been detected using cultivation-independent methods (Jackson et al., 2006). These genera belong to the class of α-Proteobacteria which is a group of gram-negative and rod-shaped bacteria. Members of rhizobia were commonly isolated from soil, and also from a wide variety of natural habitats, such as fresh water, sediments, plant roots, and plant stems. It had been reported that *Methylobacterium* could be isolated from leaf (Madhaiyan and Poonguzhali, 2014) and was the dominant microbes of FTL in the fermentation process (Zhang et al., 2020), and it imparts a range of beneficial effects on plant growth and development through various mechanisms (Madhaiyan et al., 2007). The molecular taxonomy has classified *Methylobacterium* as representing a line of descent in the α2 subgroup of the Proteobacteria (Madhaiyan et al., 2012). Studies showed a horizontal transfer of symbiotic genes from a *Bradyrhizobium* to *a Mesorhizobium* (Barcellos et al., 2007; Rogel et al., 2011). Most notably, *Ralstonia* was also identified by high-throughput sequencing in 10 samples with a relative abundance of <20%. Being a soil-borne pathogen with long incubation periods *Ralstonia* presumably contributes to its incidence.

Microbial functions were predicted by using the FUNGuild database and PICRUSt, where most of the fungal OTUs were recognized as pathotroph-saprotroph-symbiotroph, accompanied by pathotroph-saprotroph and pathotroph-symbiotroph. Crops may pose a risk of opportunistic fungal infection development in patients who are immunocompromised as the potential pathogenicity of those fungal crop contaminants cannot be disregarded (Ashley et al., 2018). The data signifies the presence of a large number of fungi; having phyto-pathogenic potential, the fungi are associated with tobacco phyllosphere during leaf senescence, for instance, the facultative necrotrophs, including *A*. *alternata* (the causal agent of brown spot) (Wang et al., 2016), *Cercospora nicotianae* that causes frogeye both on flue-cured and cigar tobacco (Fajola and Alasoadura, 1973; Zhao et al., 2020), *Phoma omnivirens* (the causal agent of black spot stalk) (Jiang et al., 2018), *Stagonosporopsis cucurbitacearum* that causes spot blight on tobacco (Wang et al., 2018), and *D. segeticola* that causes tobacco leaf spot (Xiang et al., 2020), along with the obligate biotroph, *G. cichoracearum* (the causal agent of powdery mildew) (Chen et al., 2019). Probably, those pathogens had arrived on tobacco leaves from diseased tissues in the field, whereas some could be growing either pathogenically inside the leaves or saprophytically on leaf surfaces during plant growth (Laforest-Lapointe et al., 2016). PICRUSt analysis revealed that the predominant functional pathway was metabolism function, followed by genetic and environmental information processing; moreover, the top three sub-functions were membrane transport, energy metabolism, and carbohydrate metabolism. Bacterial gene function prediction showed no significant difference among the 16 samples.

The result of substrate utilization profiling (BIOLOG) displayed that microbial communities had a strong utilization ability of carbohydrates, followed by carboxylic acids, amino acids, polymers, and amines; however, the utilization of phenolic compounds was the minimum. The substrate utilization characteristics of microbial communities in two kinds of leaf groups remained the same, which increased from the first to the third collection periods, while the healthy group had relatively higher utilization rates of carbon sources than the diseased group except for the phenolic compounds. The middle leaves had a higher utilization ability of 6 carbon sources in both leaf types, followed by the upper and lower leaves. Furthermore, a higher microbial diversity on tobacco leaves infected with tobacco brown spots during different growth stages was yielded from high-throughput sequencing than the culture-dependent method.

## Conclusion

In this study, spatiotemporal variation of the community and the divergence of phyllospheric microflora were explored to be associated with three different leaf positions across three leaf growth stages (advancing gradually toward leaf senescence) in healthy or diseased leaves. More fungal and bacterial genera were obtained by metagenomics than by the culture-dependent method. Apparently, there were some differences between healthy and diseased leaf types. The abundance of Ascomycota and Basidiomycota from diseased leaves was higher than in healthy leaves in three periods in all positions by high-throughput sequencing. Moreover, the abundance of main fungal community composition was affected by time-points, and the time-point was the main factor that could affect the fungal community composition in the diseased group. The abundance of two main fungal phyla (Ascomycota and Basidiomycota) and genera (*Alternaria* and *Phoma*) from the middle and lower positions at the last stage was much higher than samples at the first and second stages in the diseased group. Function prediction analysis visualized the microbial community structure in tobacco leaves and better mining the microbe succession in varying spatiotemporal environments. Pathotroph-saprotroph-symbiotroph was the dominant trophic mode of the fungal community and metabolism function was the predominant pathway in the three stages. Carbohydrates were the main carbon source for microbes.

## Data Availability Statement

The datasets presented in this study can be found in online repositories. The names of the repository/repositories and accession number(s) can be found at: https://www.ncbi.nlm.nih.gov/, PRJNA751254 and PRJNA751261.

## Author Contributions

Y-fD, X-mW, H-cW, and IS conceived and designed the experiment. Y-fD, L-tC, and H-cW conducted the experiment and collected the samples. H-cW and J-xL performed the analysis of samples. Y-fD and H-cW analyzed the data. Y-fD wrote the first draft of the manuscript. H-cW, X-mW, FW, SS, and IS revised the manuscript. All authors read and approved the submitted version.

## Funding

This work was supported by the National Natural Science Foundation of China (32160522, 31960550, and 32160656), the China National Tobacco Corporation [110202001035(LS-04) and 110202101048(LS-08)], the Guizhou Science Technology Foundation [ZK(2021)Key036], the International Science and Technology Cooperation Base [(2020)4102], Guizhou Provincial Academician Workstation of Microbiology and Health [(2020)4004], the Guizhou Tobacco Company (2020XM22 and 2020XM03), the Cultivation Program of Guizhou University [(2019)09], the Hundred Level Innovative Talent Foundation of Guizhou Province [GCC(2022)023-1 and GCC(2022)028-1], the Sino-Pakistan Project NSFC (31961143008), the National Natural Science Foundation of China, the International (Regional) Cooperation and Exchange Program, the Research fund for International young scientists (31750110462), and the Jiangsu Collaborative Innovation Center for Modern Crop Production (JCIC-MCP) China.

## Conflict of Interest

Y-fD was employed by Bijie Tobacco Company. J-xL was employed by Guizhou Tobacco Company of CNTC, China National Tobacco Corporation. The remaining authors declare that the research was conducted in the absence of any commercial or financial relationships that could be construed as a potential conflict of interest.

## Publisher's Note

All claims expressed in this article are solely those of the authors and do not necessarily represent those of their affiliated organizations, or those of the publisher, the editors and the reviewers. Any product that may be evaluated in this article, or claim that may be made by its manufacturer, is not guaranteed or endorsed by the publisher.
