# Supplementary material for: Spatio-Temporal Variation in the Phyllospheric Microbial Biodiversity of Alternaria Alternata-Infected Tobacco Foliage
Source: Front Microbiol. 2022 Jul 28;13:920109. doi: 10.3389/fmicb.2022.920109 (PMC9370072; doi:10.3389/fmicb.2022.920109)
Supplement: Supplementary file 1 [file Data_Sheet_1.pdf]

Spatio-temporal variation in the phyllospheric microbial biodiversity of *Alternaria alternata* infected tobacco foliage

Yuanfeng Dai [1, 2, 3], Xiaomao Wu [1, *], Hancheng Wang [2, *], Wenhong Li [4], Liuti Cai [2], Jixin Li [5], Feng Wang [2, *], Shafaque Sehar [6], Imran Haider Shamsi [6, *]

[1]*College of Agriculture, Guizhou University, Guiyang 550025, Guizhou, China;*

[2]*Guizhou Provincial Academician Workstation of Microbiology and Health, Guizhou Academy of Tobacco Science, Guiyang 550081, China*

[3]*Bijie Tobacco Company, Bijie 551700, China*

[4]*Guizhou Institute of Plant Protection, Guizhou Academy of Agricultural Sciences, Guiyang 550025, China*

[5]*Guizhou Tobacco Company of CNTC, China National Tobacco Corporation, Guiyang 550001, China*

[6]*Zhejiang Key Laboratory of Crop Germplasm Resource, Department of Agronomy, College of Agriculture and Biotechnology, Zhejiang University, Hangzhou, 310058, China*

*Corresponding author's email: Prof. Xiaomao Wu (wuxm827@126.com), Prof. Hancheng Wang (xiaobaiyang126@hotmail.com), Prof. Wang Feng (yancaowangfeng@163.com), and Prof. Imran Haider Shamsi (drimran@zju.edu.cn)

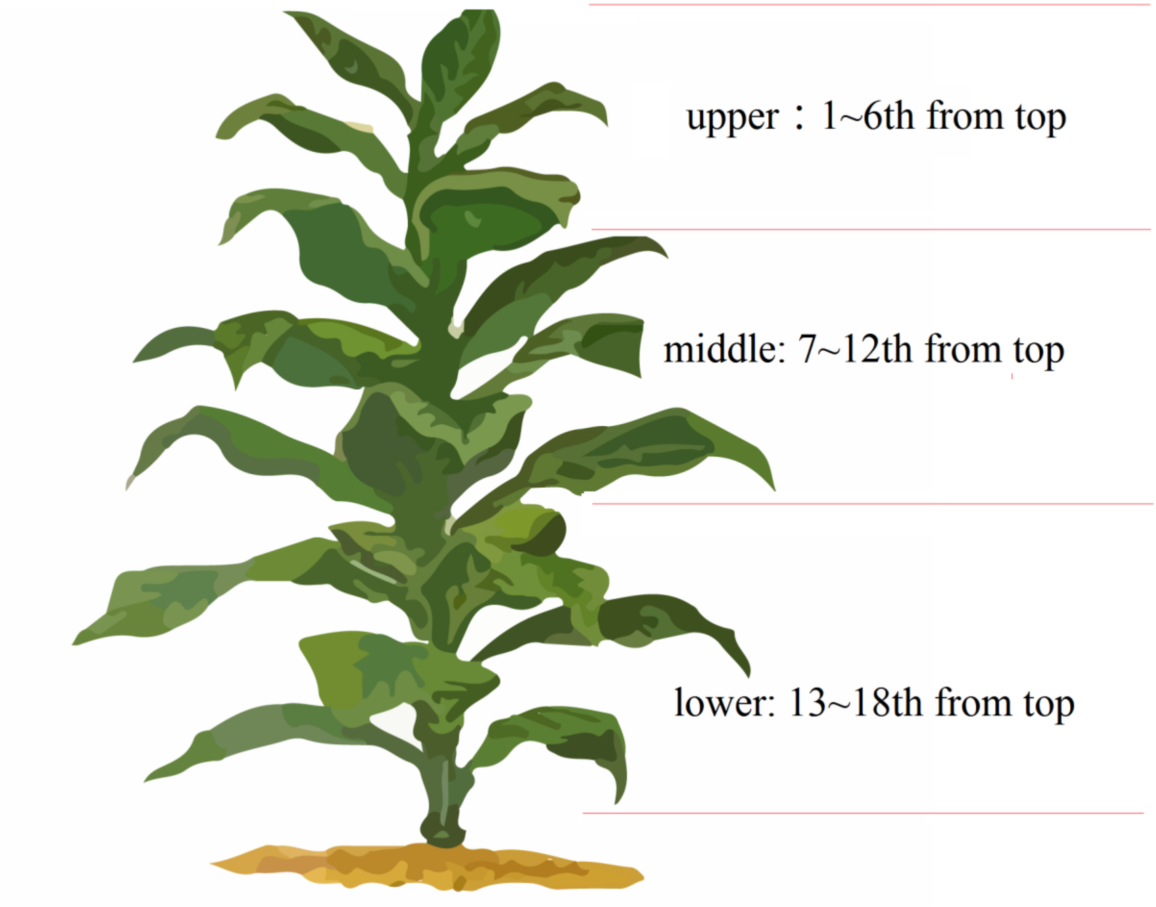

Figure S1. Sample names were shown schematically. leaf position upper (1~6th top leaf), middle (7~12th top leaf) and lower (13~18th top leaf) named from the top of flue-cured tobacco plant.

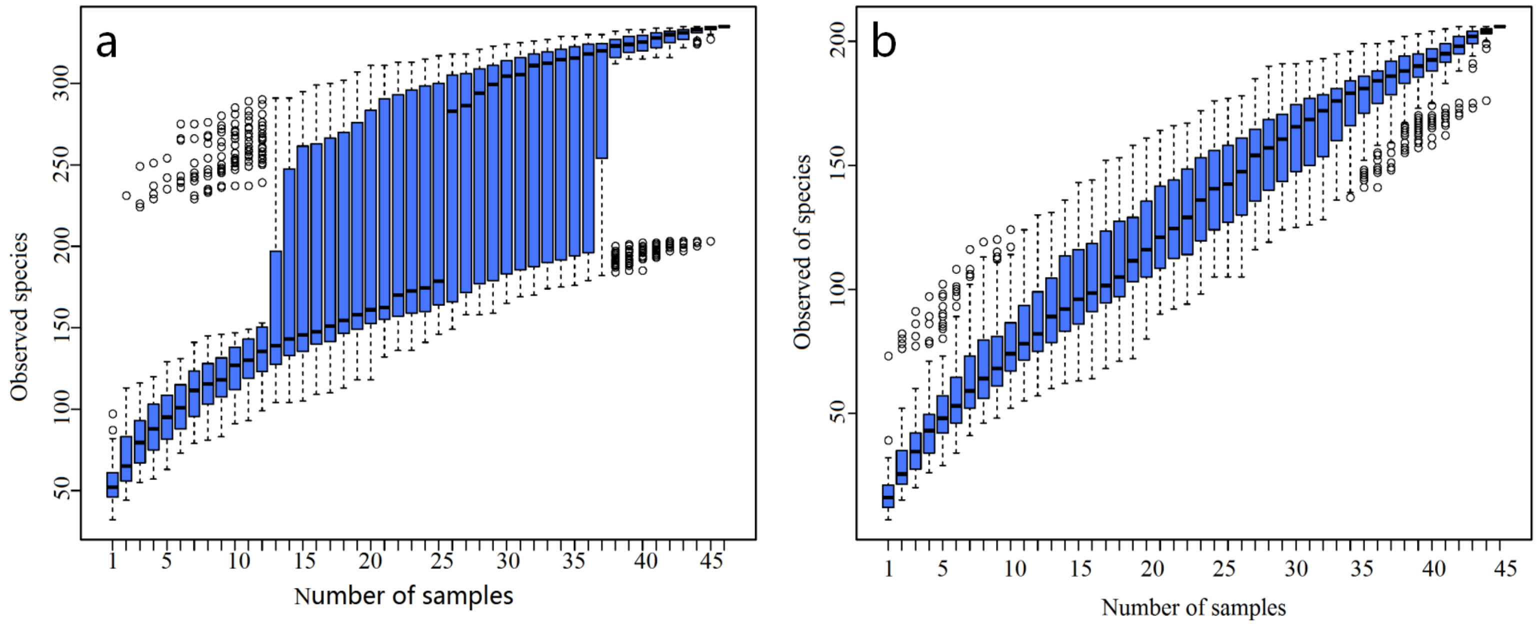

Figure S2. Species accumulation boxplot of different tobacco leaf samples（a: fungi, b: bacteria）

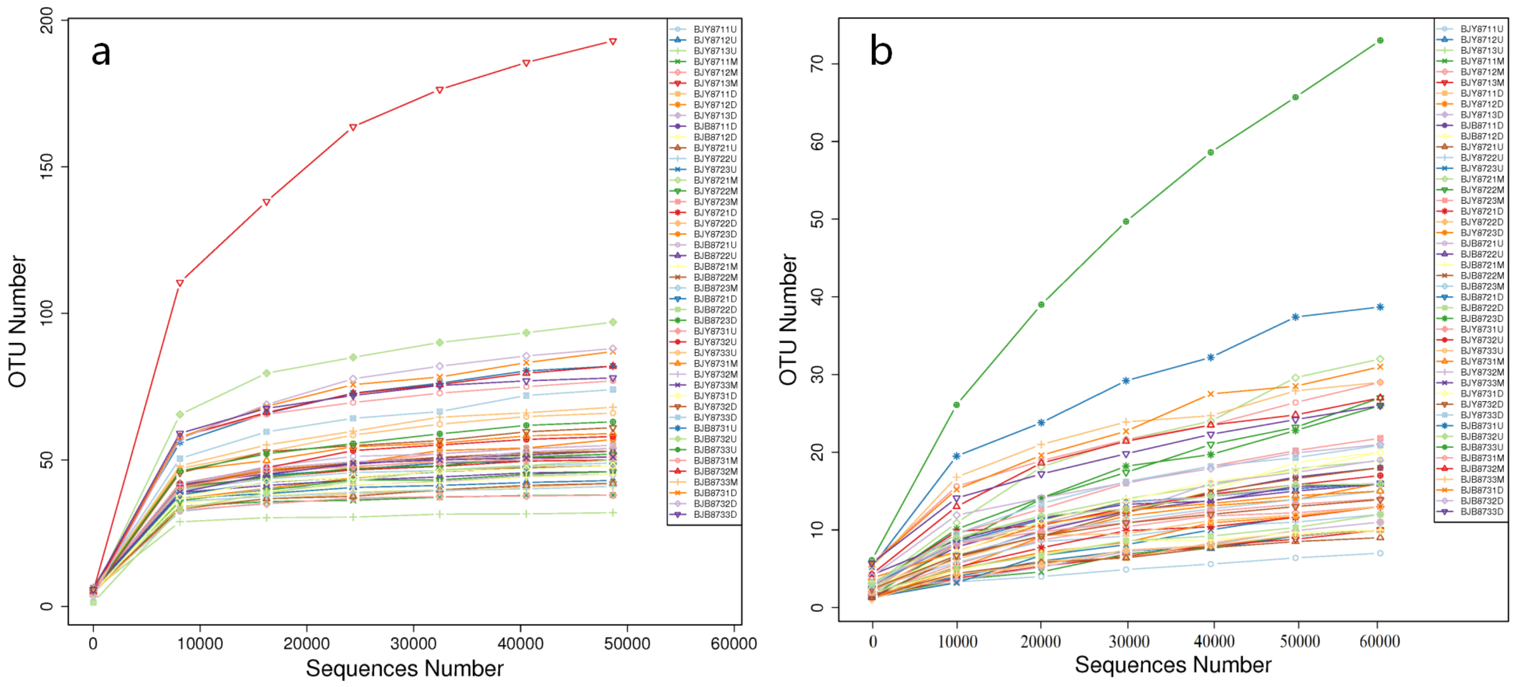

Figure S3. Rarefaction curves of OTUs across different tobacco leaf samples（a: fungi, b: bacteria）

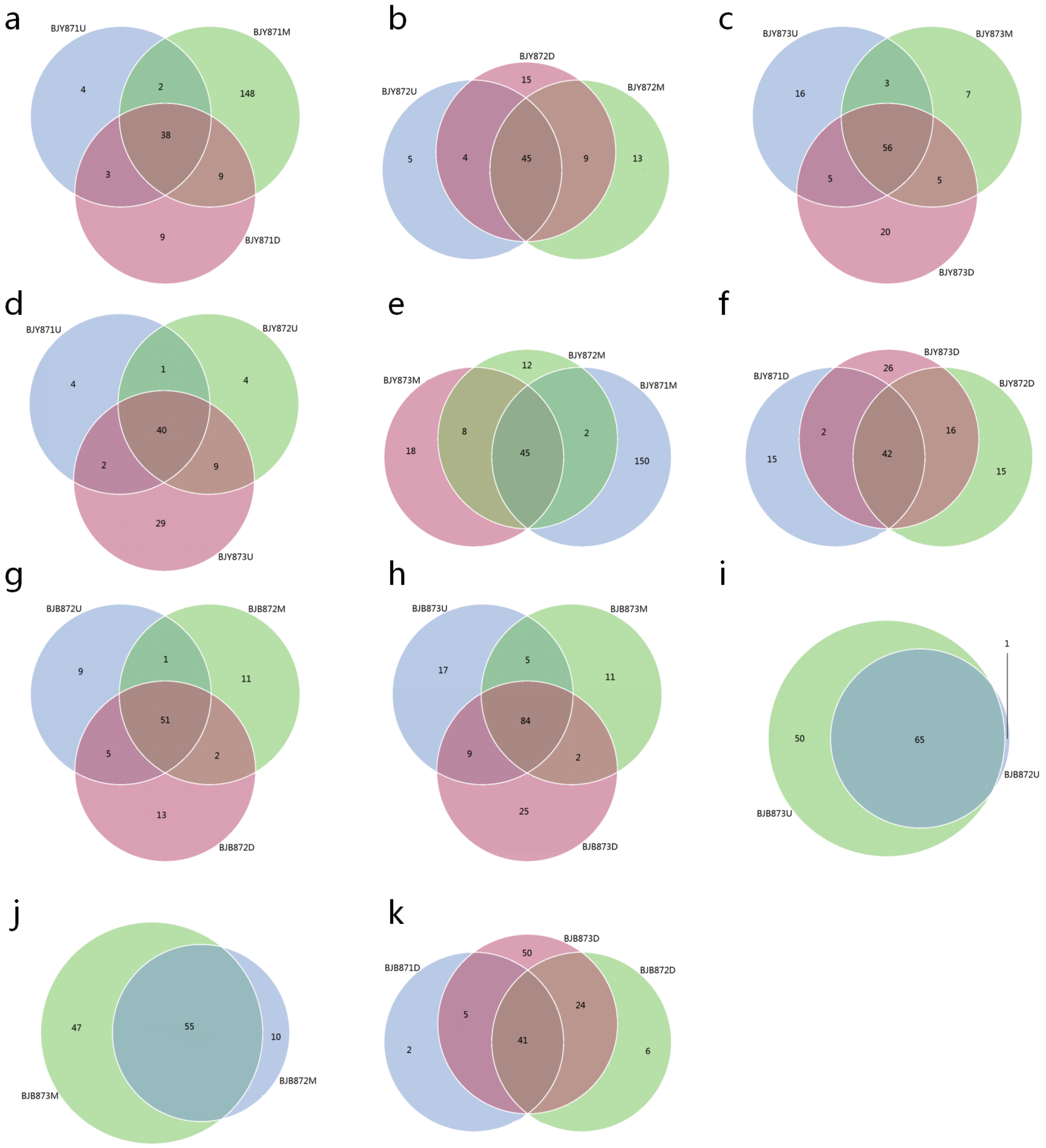

Figure S4. Venn diagram illustrating the fungal OTUs count as detected in healthy and diseased tobacco leaves. Fungal groups in healthy leaves from three different position (a-c) and from the same position (d-f) at three time. Fungal groups diseased leaves from three different position (g-h) at the second and third time and from the same position (i-k) at three time.

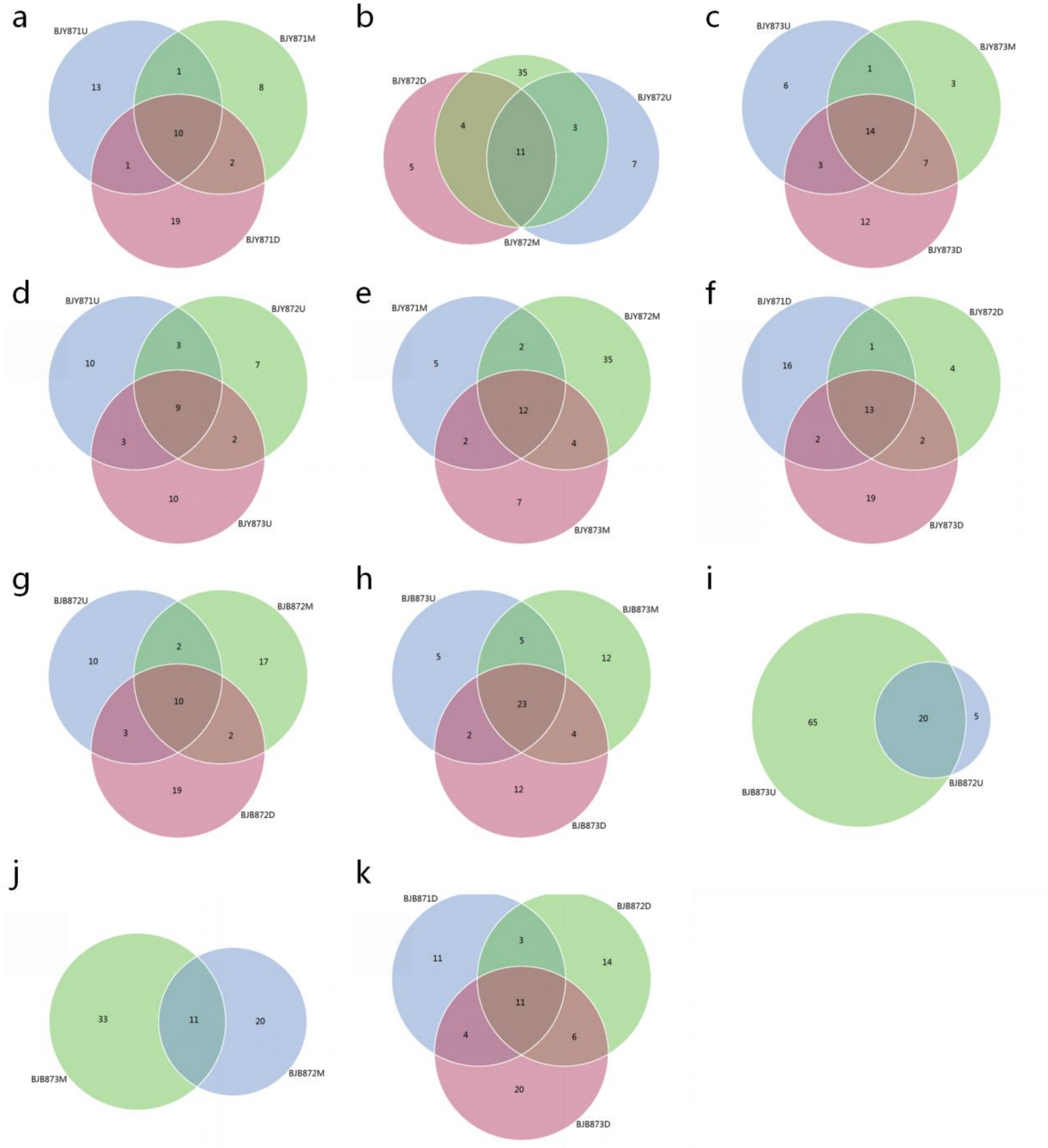

Figure S5. Venn diagram displaying the bacterial OTU count as detected in healthy and diseased tobacco leaves. Bacterial OTUs in healthy groups from three different position (a-c) and from the same position (d-f) at three time. Bacterial OTUs in diseased groups from three different position (g-h) at the second and third time and from the same position (i-k) at three time.

Table S1. Detailed information of samples.

Time for collecting	Plant position	Sample name	
		Healthy leaves	Diseased leaves
First time (2020.8.8)	1-6th top leaf (upper)	BJY871U	
	7-12th top leaf (middle)	BJY871M	
	13-18th top leaf (lower)	BJY871D	BJB871D
Second time (2020.8.30)	1-6th top leaf (upper)	BJY872U	BJB872U
	7-12th top leaf (middle)	BJY872M	BJB872M
	13-18th top leaf (lower)	BJY872D	BJB872D
Third time (2020.9.19)	1-6th top leaf (upper)	BJY873U	BJB873U
	7-12th top leaf (middle)	BJY873M	BJB873M
	13-18th top leaf (lower)	BJY873D	BJB873D

Table S2. Molecular identification of the total leaf fungi and bacteria isolated from healthy and diseased tobacco leaves where tobacco brown spot disease occurred.

Collection time	Media	No. of Species	Species	Strain code (GenBank No.)	Position			leaf		Total (% of fungal or bacterial isolates)
					Up	Middle	Lower	Healthy	Diseased	
**Fungi**										
The first time	AEA	11	*Cercophora* sp.	1A1（MZ408895）,1A2（MZ408896）,1A3（MZ408897）,1A4（MZ408898）, 17A2（MZ413252）	4		1	4	1	5(3.27%)
			Coprinopsis sp.	3A3（MZ413121）			1	1		1(0.65%)
			Fusarium sp.	10A6（MZ413179）			1		1	1(0.65%)
			Penicillium sp.	10A2（MZ413234）			1		1	1(0.65%)
			Xylariales sp.	2A（MZ413227）		1		1		1(0.65%)
			Trametes sp.	3A1（MZ408901）			1	1		1(0.65%)
			Didymellaceae sp.	3A1-1（MZ413188）			1	1		1(0.65%)
			Paraphoma sp.	3A2（MZ413182）			1	1		1(0.65%)
			Sordariomycetes sp.	3A5（MZ413122）			1	1		1(0.65%)
			Bjerkandera sp.	10A1（MZ413229）			1		1	1(0.65%)
			Coprinopsis sp.	17A1（MZ413163）			1		1	1(0.65%)
	PDA	8	*Cercophora* sp.	3P（MZ413123),17P1（MZ413164）,17P2（MZ413175),17P2-1（MZ413166), 17P3（MZ413165）,17P3-3（MZ413167）			6	1	5	6(3.92%)
			Coprinopsis sp.	2P1（MZ408899）		1		1		1(0.65%)
			Fusarium sp.	10P4（MZ413191）			1	1		1(0.65%)
			Penicillium sp.	10P1（MZ413233）			1	1		1(0.65%)
			Alternaria sp.	10P6（MZ413177）,10P3-2（MZ413183）			2		2	2(1.31%)
			Daldinia sp.	19P1（MZ413240）	1			1		1(0.65%)
			Nemania sp.	2P2（MZ408900）		1		1		1(0.65%)
			Zopfiella sp.	17P4（MZ413189）			1	1		1(0.65%)
The second time	AEA	16	*Alternaria* sp.	4A2(MZ413228),4A4(MZ413190),4A5(MZ413126), 5A3(MZ413132),6A4(MZ413143),112A9(MZ413245),18M1(MZ413186)	3	2	2	5	2	7(4.58%)
			Cercophora sp.	6A3(MZ413142),6A5(MZ413144),24A8(MZ413202),111A6(MZ413243)	2		2	2	2	4(2.61%)
			Botrytis sp.	5A1(MZ413130),5A5(MZ413134),4A1(MZ413124),6A6(MZ413145)	1	2	1	4		4(2.61%)
			Coprinopsis sp.	5A2(MZ413131),24A4(MZ413215),113A1(MZ413247),113A10(MZ413249)	1	1	2	1	3	4(2.61%)
			Didymella sp.	5A4(MZ413133),6A1(MZ413140),13A5(MZ413238)		1	2	1	2	3(1.96%)
			Mucor sp.	112A1(MZ413226),12A1(MZ413235)		2			2	2(1.31%)

			Paraphoma sp.	6A2(MZ413141),12A2(MZ413232)		1	1	1	1	2(1.31%)
			Verticillium sp.	24A5(MZ413216)	1				1	1(0.65%)
			Gibellulopsis sp.	24A6(MZ413200)	1				1	1(0.65%)
			Cladosporium sp.	4A3(MZ413125),24A7(MZ413201)	2			1	1	2(1.31%)
			Cadophora sp.	12A3(MZ413192)		1			1	1(0.65%)
			Daldinia sp.	13A4(MZ413237)			1		1	1(0.65%)
			Preussia sp.	113A4(MZ413248)			1		1	1(0.65%)
	PDA	15	*Alternaria* sp.	4P2(MZ413128),4P3(MZ413129),112P5(MZ413246)	2	1		2	1	3(1.96%)
			Botrytis sp.	5P2(MZ413136),5P3(MZ413137),5P4(MZ413138),5P5(MZ413139),6P3(MZ413147)		4	1	5		5(3.27%)
			Didymella sp.	5P1(MZ413135),113P1(MZ413250)，13P2(MZ413154)		1	2	1	2	3(1.96%)
			Cercophora sp.	4P1(MZ413127)	1			1		1(0.65%)
			Paraphoma sp.	113P3(MZ413255),13P9(MZ413193)			2		2	2(1.31%)
			Mucor sp.	112P2(MZ413203)，113P2(MZ413204)		1	1		2	2(1.31%)
			Fusarium sp.	6P1(MZ413146)			1	1		1(0.65%)
			Irpex sp.	111P1(MZ413244)	1				1	1(0.65%)
			Cladosporium sp.	111P2(MZ413222)	1				1	1(0.65%)
			Chaetomium sp.	113P2-1(MZ413223)			1		1	1(0.65%)
			Coprinopsis sp.	113P4(MZ413251)			1		1	1(0.65%)
			Brunnipila sp.	113P6(MZ413256)			1		1	1(0.65%)
The third time	AEA	23	*Alternaria* sp.	18M4(MZ413185),18M8(MZ413176)		1	1	2		2(1.31%)
			Coprinopsis sp.	25A1(MZ413217),25A11(MZ413221)，25A10(MZ413254), 8A1(MZ413149), 8A3(MZ413151) ,8A4(MZ413152),116A1-2(MZ413197)		6	1	6	1	7(4.58%)
			Cercophora sp.	8A5(MZ411672),9A(MZ413153),15A1(MZ413158), 16A2(MZ413162),16A5(MZ413195)		2	3	2	3	5(3.27%)
			Nemania sp.	20A6(MZ413207)		1			1	1(0.65%)
			Paraphoma sp.	22A4(MZ413199)	1			1		1(0.65%)
			Schizophyllum sp.	25A5(MZ413218)		1		1		1(0.65%)
			Botrytis sp.	25A7(MZ413219)		1		1		1(0.65%)
			Cladosporium sp.	25A8(MZ413220)		1		1		1(0.65%)
			Peniophora sp.	25A9(MZ413253)		1		1		1(0.65%)
			Coprinellus sp.	8A2(MZ413150)		1		1		1(0.65%)
			Cadophora sp.	21A3(MZ413260)			1	1		1(0.65%)
			Bjerkandera sp.	21A4(MZ413212)			1	1		1(0.65%)

			Mucor sp.	14A11(MZ413155),15A2(MZ413239),15A5(MZ413160)	1	2			3	3(1.96%)
			Pestalotiopsis sp.	18M3(MZ413174)	1				1	1(0.65%)
			Aspergillus sp.	18M5(MZ413187)	1				1	1(0.65%)
			Penicillium sp.	15A3(MZ413159)，116A5(MZ413224)		1	1		2	2(1.31%)
			Daldinia sp.	20A3(MZ413205)		1			1	1(0.65%)
			Trametes sp.	20A5(MZ413206)		1			1	1(0.65%)
			Chaetomium sp.	16A3(MZ413241)			1		1	1(0.65%)
	PDA	24	*Coprinopsis* sp.	8P1(MZ413171),25P7(MZ413265),116P3(MZ413225)，9P1(MZ413178),25P1(MZ413261),18P1(MZ413198)	1	3	2	4	2	6(3.92%)
			Cercophora sp.	15P1(MZ413180),20P3(MZ413196)		2			2	2(1.31%)
			Alternaria sp.	9P2(MZ413230),14P8(MZ413157)	1		1	1	1	2(1.31%)
			Nemania sp.	15P3(MZ413181),20P3-1(MZ413209),20P5(MZ413210), 20P6(MZ413211),116P6(MZ413258), 116P7(MZ413259)，21P4(MZ413213)		4	3	1	6	7(4.58%)
			Fusarium sp.	18Pc(MZ413172),18PE(MZ413173),18P2-4(MZ413242)	3				3	3(1.96%)
			Cadophora sp.	14P4(MZ413194),25P4(MZ413264)	1	1		1	1	2(1.31%)
			Coniochaeta sp.	25P2(MZ413262)		1		1		1(0.65%)
			Coprinellus sp.	25P3(MZ413263)		1		1		1(0.65%)
			Aspergillus sp.	9P3(MZ413236)			1	1		1(0.65%)
			Hypoxylon sp.	21P5(MZ413214)			1	1		1(0.65%)
			Pestalotiopsis sp.	7P8(MZ413148)	1			1		1(0.65%)
			Myrmaecium sp.	8P2(MZ413170)		1		1		1(0.65%)
			Didymella sp.	14P7(MZ413184)	1				1	1(0.65%)
			Mucor sp.	7P1(MZ413231),14P1(MZ413156)，15P2(MZ413161),16P2(MZ413168)	2	1	1	1	3	4(2.61%)
			Rhizopus sp.	16P3(MZ413169)			1		1	1(0.65%)
			Xylaria sp.	20P2(MZ413208),116P5(MZ413257)		1	1		2	2(1.31%)
**Bacteria**										
The first time	NA	7	*Pseudomonas* sp.	1N(MZ377148),3N1-2(MZ377150),2N(MZ377152), 3N3(MZ377153)	1	1	2	4		4(4.04%)
			Variovorax sp.	19N3(MZ377155),19N3-1(MZ377192),19N2-3(MZ377193)	3			3		3(3.03%)
			Brevundimonas sp.	19N4(MZ377194),19N1(MZ377228)	2			2		2(2.02%)
			Leclercia sp.	3N1-1(MZ377229)			1	1		1(1.01%)
			Acidovorax sp.	19N2(MZ377222)	1			1		1(1.01%)
			Stenotrophomonas sp.	3N2-2(MZ377154)			1	1		1(1.01%)
	LB	9	*Pseudomonas* sp.	1L(MZ377149),2L(MZ377151),3L1(MZ377156)	1	1	1	3		3(3.03%)

			Variovorax sp.	19L3(MZ377195),19L2(MZ377225)	2			2		2(2.02%)
			Acidovorax sp.	19L6(MZ377202),19L5(MZ377223)	2			2		2(2.02%)
			Enterobacter sp.	3L2(MZ377231)			1	1		1(1.01%)
			Bacillus sp.	10L2(MZ377234)			1		1	1(1.01%)
			Brevundimonas sp.	19L4(MZ377226),19L4-1(MZ377196)	2			2		2(2.02%)
			Pantoea sp.	19L1-1(MZ377237)	1			1		1(1.01%)
The second time	NA	8	*Pseudomonas* sp.	4N2(MZ377157),5N2-1(MZ377158),11N2(MZ377299)，5N1(MZ377212)	2	2		3	1	4(4.04%)
			Pantoea sp.	11N1-2(MZ377159),11N1(MZ364018),11N1-5(MZ364019)，11N3(MZ377176)	4				4	4(4.04%)
			Microbacterium sp.	12N1(MZ377235)		1			1	1(1.01%)
			Sphingomonas sp.	12N2(MZ377227)，13N2(MZ377209)		1	1		2	2(2.02%)
	LB	5	*Pseudomonas* sp.	4L(MZ377204),4L1(MZ377197),5L1(MZ377160)，6L1(MZ377161)	2	1	1	4		4(4.04%)
			Pantoea sp.	11L1(MZ377077),11L2(MZ377078),11L3(MZ377145), 11L4(MZ377146),11L4-3(MZ377208),13L1(MZ377236)	5		1		6	6(6.06%)
			Microbacterium sp.	12L1(MZ377210)		1			1	1(1.01%)
			Sphingomonas sp.	12L2(MZ377211)		1			1	1(1.01%)
The third time	NB	12	*Pantoea* sp.	8N1(MZ377205),8N1-1(MZ377206),14N1(MZ377177),14N1-2(MZ377203), 14N3(MZ377179), 18N1-1(MZ377200), 18N1-3(MZ377201), 18N4(MZ377147),15N1(MZ377184), 16N1-1(MZ377188),16N2(MZ377215),16N1(MZ377186)	6	3	3	2	10	12(12.12%)
			Pseudomonas sp.	7N3(MZ377232),8N2(MZ377233),9N1(MZ377172),9N1-1(MZ377173),14N2(MZ377213)	2	1	2	4	1	5(5.05%)
			Bacillus sp.	7N1(MZ377162)	1			1		1(1.01%)
			Luteibacter sp.	7N2(MZ377163)	1			1		1(1.01%)
			Stenotrophomonas sp.	14N2-1(MZ377178)	1				1	1(1.01%)
			Curtobacterium sp.	18N3-3(MZ377217)	1				1	1(1.01%)
			Microbacterium sp.	18N2(MZ377190)	1				1	1(1.01%)
			Xanthomonas sp.	18N3(MZ377191)	1				1	1(1.01%)
	LB	19	*Brevundimonas* sp.	7L1(MZ377164),8L1-1(MZ377207)	1	1		2		2(2.02%)
			Pantoea sp.	8L1(MZ377168),8L3-1(MZ377171),14L3(MZ377183),14L2(MZ377181),14L2-1(MZ377182),14L3-5(MZ377199),15L1(MZ377185),16L1-1(MZ377187),16L2(MZ377216)	4	3	2	2	7	9(9.09%)

Sphingomonas sp.	7L4(MZ377167)	1			1		1(1.01%)
Stenotrophomonas sp.	14L1(MZ377180),14L1-1(MZ377198)	2				2	2(2.02%)
Curtobacterium sp.	18L3-1(MZ377219),18L3-2(MZ377220),18L3(MZ377230),7L3(MZ377166)	4			1	3	4(4.04%)
Pseudomonas sp.	8L2(MZ377169),8L2-1(MZ377170), 9L1(MZ377174), 9L2(MZ377175),14L4(MZ377214),16L1(MZ377189)	1	2	3	4	2	6(6.06%)
Agrobacterium sp.	16L2-1(MZ377298)			1		1	1(1.01%)
Bacillus sp.	16Lc(MZ377224)			1		1	1(1.01%)
Chryseobacterium sp.	18L4(MZ377221)	1				1	1(1.01%)
Luteibacter sp.	7L2(MZ377165)	1			1		1(1.01%)
Xanthomonas sp.	18L2(MZ377218)	1				1	1(1.01%)
